# Evaluation of *TGFBI* corneal dystrophy and molecular diagnostic testing

**DOI:** 10.1038/s41433-019-0346-x

**Published:** 2019-02-13

**Authors:** Connie Chao-Shern, Lawrence A. DeDionisio, Jun-Heok Jang, Clara C. Chan, Vance Thompson, Kathleen Christie, M. Andrew Nesbit, C. B. Tara McMullen

**Affiliations:** 1 Biomedical Sciences Research Institute, University of Ulster, Coleraine, Northern Ireland UK; 2 Avellino Lab USA, Inc., Menlo Park, CA USA; 3 Department of Ophthalmology, University of Toronto, Toronto, Canada; 4 Vance Thompson Vision, Sioux Falls, SD USA

**Keywords:** Mutation, Corneal diseases

## Abstract

To date, 70 different TGFBI mutations that cause epithelial-stromal corneal dystrophies have been described. At present one commercially available test examines for the five most common of these mutations: R124H, R124C, R124L, R555W, and R555Q. To expand the capability of identifying the causative mutation in the remaining cases, 57 mutations would need to be added. The aim of this study was to obtain a better understanding of the worldwide distribution and population differences of TGFBI mutations and to assess which mutations could be included or excluded from any potential assay. A total of 184 published papers in Human Gene Mutation Database (HGMD) and PubMed from 34 countries worldwide reporting over 1600 corneal dystrophy cases were reviewed. Global data from 600,000 samples using the commercially available test were analyzed. Case studies by University College of London (UCL), Moorfield’s Corneal Dystrophy Study data and 19 samples from patients with clinical abnormality or uncertainty for which the current test detected no mutation were used to predict an achievable detection rate. Data from the literature search showed no difference in the spectrum and frequency of each mutation in different populations or geographical locations. According to our analysis, an increase to the worldwide detection rate in all populations from 75 to 90% could be achieved by the addition of six mutations—H626R, A546D, H572R, G623D, R124S, and M502V—to the currently available test and that may be beneficial for LASIK pre-screening worldwide.

## 摘要

到目前为止, 已经发现62种不同的TGFBI突变导致角膜上皮间质性营养不良。商业化的检测方法用来检测这些突变中最常见的5个突变位点:R124H、R124C、R124L、R555W和R555Q。为了扩大此基因在病例中识别致病突变的能力, 需要添加57个突变。本研究的目的是为了更好地了解TGFBI突变在世界范围内的分布和种群差异, 并评估哪些突变可以被纳入或排除在任何潜在的分析中。来自全球34个国家的184篇发表在人类基因突变数据库(HGMD)和PubMed上的论文报道了超过1600例角膜营养不良病例。研究人员分析了来自全球60万个样本的数据, 这些样本使用的是市面上可以买到的检测方法。来自伦敦大学学院(UCL)、Moorfield’s角膜营养不良的病例研究数据和目前检测未发现突变的19例临床异常或不确定的患者样本进行分析, 预测出可获得的检出率。文献检索的数据显示, 在不同种群或地理位置, 每个突变的频谱和频率没有差异。根据我们的分析, 在现有的检测方法基础上, 通过增加H626R、A546D、H572R、G623D、R124S和M502V六种突变, 可以将全球所有人群的检出率从75%提高到90%, 这将有利于全世界LASIK的预先筛分。

## Introduction

The cornea is an avascular transparent tissue at the front of the eye that begins the process of focusing light onto the retina and accounts for around two-thirds of the eye’s optical power. A number of heritable conditions affect corneal clarity, and they are categorized by the affected corneal layer as posterior, stromal or superficial [1]. Autosomal dominant (AD), X-linked recessive (XR), and autosomal recessive (AR) inheritance patterns have all been observed, and in many cases, the disease locus has been mapped and the causative gene has been identified. The most studied corneal dystrophies are those caused by AD missense mutations in the transforming growth factor beta-induced gene (*TGFBI*) located on chromosome 5q31.1, which encodes an extracellular matrix protein thought to play pivotal roles in physiologic and pathologic responses by mediating cell adhesion, migration, proliferation and differentiation [2]. To date, 70 *TGFBI* mutations are reported in the Human Gene Mutation Database (HGMD) to cause a spectrum of different epithelial-stromal corneal dystrophies with corneal amyloid and non-amyloid deposits, including granular corneal dystrophy type 1 (GCD1) and type 2 (GCD2, previously designated as Avellino Corneal Dystrophy [3]), epithelial basement membrane dystrophy (EBMD), lattice corneal dystrophy (LCD), Reis-Bücklers corneal dystrophy (RBCD) and Thiel-Behnke corneal dystrophy (TBCD) [4, 5]. Different *TGFBI* mutations can cause specific corneal dystrophies, and a genotype-phenotype correlation has been demonstrated at two mutation hotspots, R124 and R555 [5].

Laser in situ keratomileusis (LASIK) is a surgical procedure that provides vision correction for myopia (nearsightedness), hyperopia (farsightedness), and astigmatism. A thin flap in the corneal epithelium and anterior stroma is cut and folded, and the exposed stromal layer is reshaped by laser to change its corneal focusing power. Photorefractive keratectomy (PRK) and phototherapeutic keratectomy (PTK) surgery affect vision correction or treat various ocular disorders by removing superficial opacities and surface irregularities from the cornea. These surface corneal surgeries induce a wound in the stromal layer, which causes the expression of *TGFBI* to be upregulated, resulting in corneal amyloid deposition within the corneas of individuals who carry the *TGFBI* mutations leading to pathology associated with corneal dystrophy [6]. It is known that GCD1, LCD1, RBCD, and TBCD have early childhood onsets. However, GCD2 carries a different presentation. The initial age of onset is dependent on whether the patient is heterozygous or homozygous for the mutation [7]. Homozygous patients are diagnosed as early as 3 years, while heterozygous have a delayed presentation. Given the delay in presentation of heterozygote, some of these patients have undergone laser vision correction. Unfortunately, many reports have demonstrated the exacerbation of GCD2 after treatment with PRK, LASIK, and PTK. Consequently, LASIK is contraindicated in GCD2 [7]. Therefore, it is our opinion that genetic screening for these late onset, heterozygous mutations should be performed before refractive surgeries [6–11]. A commercially available genetic test has been developed that can detect within the *TGFBI* gene the five most common mutations which are linked to the five more common types of corneal dystrophy.

- R124H for granular corneal dystrophy type 2
- R124C for lattice corneal dystrophy type 1
- R124L for Reis-Buckler corneal dystrophy
- R555W for granular corneal dystrophy type 1
- R555Q for Thiel-Behnke corneal dystrophy

This five-mutation genetic test was originally designed for the Korean and Japanese population, where a majority of the *TGFBI* corneal dystrophy cases are diagnosed as GCD2 caused by the R124H mutation [12]. Within Korea and Japan, the test is used primarily as a screening tool prior to refractive surgery. However, in the US and Europe, the test is used both to screen refractive surgery candidates and as a confirmatory test for clinical diagnosis of corneal dystrophy disease. The purpose of this study is to review the prevalence of different *TGFBI* mutations in various populations and geographic locations to determine whether the available genetic test, as currently constituted, is optimal for use in different populations worldwide.

## Materials and methods

### Worldwide literature search

A worldwide literature search was performed using the articles curated in the HGMD database (QIAGEN, Hilden, Germany) via a paid academic research version, last accessed on 23 February 2018 and articles in PubMed (US National Library of Medicine, National Institutes of Health). Reviewed herein are 184 articles with over 1600 reported individual patient cases (Supplementary Material). An interactive world map based on the data within the literature and developed with a Google Maps application was created and used to plot the reported mutation information, ethnicities, and case numbers (a copy of the link is available upon request).

### Global available genetic test data analysis

The available genetic test (Avellino Labs USA, Menlo Park, CA) was utilized to test over 600,000 patient samples worldwide (Korea, Japan, China, USA, and Europe). In short, epithelial cells were collected from subjects’ buccal mucosa with Copan buccal swabs (Copan Italia, Brescia, and Italy), which were subsequently inserted into a protective outer tube. DNA extractions were carried out either with the DNA Extract All Reagents Kit or the ChargeSwitch gDNA Normalized Buccal Cell Kit (Thermo Fisher Scientific, Waltham, MA, USA). DNA amplification was produced by TaqMan GTXpress Master Mix or TaqPath ProAMp Multiplex Master Mix (Thermo Fisher Scientific, Waltham, MA, USA). Custom TaqMan® Assay Design Tool (ThermoFisher Scientific, Waltham, MA, USA) was used to design PCR primers and probes for each mutation, and the Custom TaqMan® Assays were manufactured by Thermo Fisher Scientific (Waltham, MA, USA). Genotyping data was collected with a 7500 FAST Real-Time PCR System (Thermo Fisher Scientific, Waltham, MA, USA). Short oligos containing wild-type and mutant sequences were utilized as control materials (ThermoFisher Scientific, Waltham, MA, USA).

### Assessment of an expanded panel with six additional mutations

Six mutations were identified from the literature search as a group of mutations with the next highest number of reported cases that may be included in an expanded testing panel. Primer and probe sets were designed using Thermo Fisher’s Custom TaqMan® Assay Design Tool (Thermo Fisher Scientific, Waltham, MA, USA). Genetic testing was conducted on epithelial cells collected from the inner cheeks with an iSWAB collection kit (Mawi DNA Technologies, Hayward, CA, USA). Genomic DNA was extracted with a QIAGEN QIAamp® DNA blood mini kit (Hilden, Germany), and whole exome sequencing (WES) was carried out with the ACE platform™ (Personalis Inc., Menlo Park, CA, USA).

Informed consent was obtained from the subjects and WES was performed on two related patients with lattice-like corneal erosions before this study was initiated. Three epithelial erosion in the lattice-like change in the center of the right eye of the 27-year-old male proband where a tree branch injury took place four years prior. The proband’s mother was subsequently examined and observed lattice-like lines on both LASIK flaps where she had bilateral LASIK surgeries 14 years previously. To determine whether there was a genetic component of the symptoms, samples were sent to us for genetic study. Real-time PCR test was designed according to the WES results for the mutation detected from both the proband and his mother. Same test designing method was used for the other five mutations. Then PCR tests were performed on the two related patients and other 17 nonrelated patients with clinical abnormality or uncertainty for which the available genetic test detected no mutation. The expanded eleven mutation panel (Table 1) was used to assess the detection rate that would have been achieved with the cohort *TGFBI* dystrophy patients in the study conducted in 2016 by University College London, Moorfield Eye Hospital [5].

**Table 1:** This table ranks the five most common mutations within reported cases (Supplementary Material) from highest to lowest. In addition, it lists the case numbers from high to low for the six additional mutations

Mutations	Reported case numbers
*Five most common mutations In the current genetic test panel*	
R124C Lattice Corneal Dystrophy type 1	372
R555W Granular Corneal Dystrophy type 1	338
R124H Granular Corneal Dystrophy type 2	325
R124L Reis-Buckler corneal dystrophy	110
R555Q Thiel-Behnke corneal dystrophy	75
*Six additional mutations In the expended test panel*	
H626R Lattice Corneal Dystrophy subtype I/IIIA	117
A546D Variant Lattice Corneal Dystrophy	48
H572R Lattice Corneal Dystrophy subtype 1	34
G623D Variant Reis-Buckler Corneal Dystrophy	26
R124S Subtype Granular Corneal Dystrophy type 1	18
M502V Variant Corneal Dystrophy and Variant Thiel-Behnke Corneal Dystrophy	4

## Results

### Worldwide literature search

**Fig. 1:**
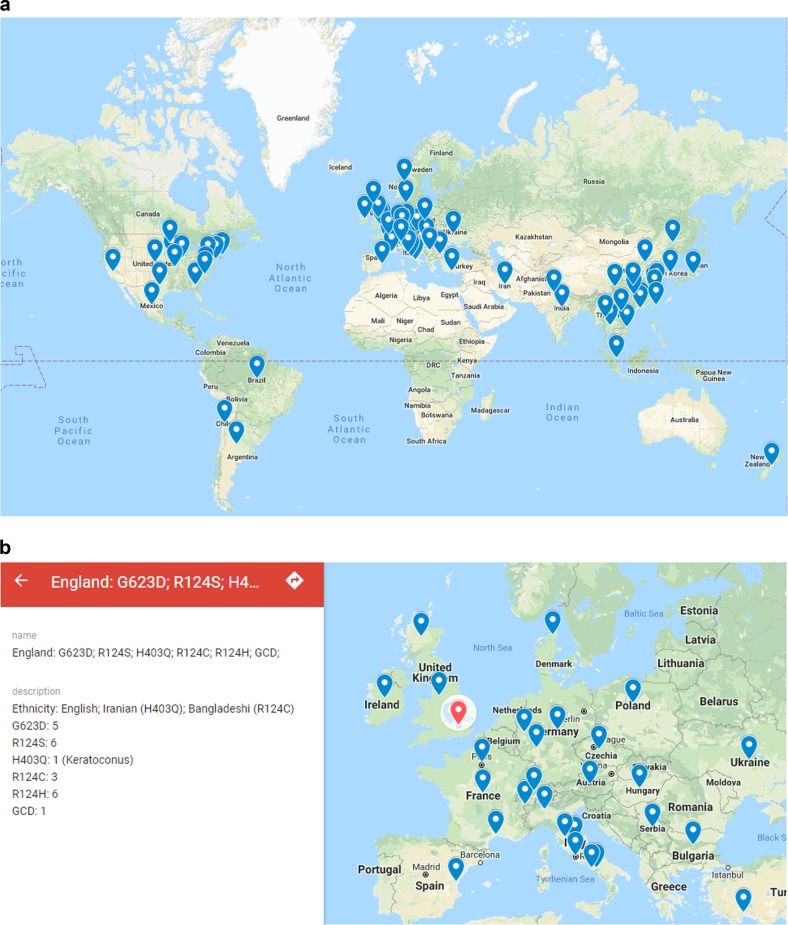
**a** World map of reported cases with various *TGFBI* mutations. Each bubble placed over a region or country contains the reported case information, such as ethnicities, mutations, and case numbers. The map illustrates that *TGFBI* mutations cases are reported all over the world, except for in regions with limited research capacity or language difficulties for publication. Very few cases were reported from South America, and no case. reports were identified from Africa or Russia. **b** The red bubble points at London, England as an example of the information contained within the bubble. The legend on the left shows the reported mutations, ethnicity and total case numbers for each reported mutation

The HGMD database was interrogated and 70 different *TGFBI* mutations were found. The HGMD database was used to identify the papers in which these mutations were described in order to build up a picture of a worldwide distribution (Fig. 1a, b). Each flag in the world map contains a summary of the mutations reported in a specific region or a country. The summary includes ethnicities, mutations and the total number of cases reported for each mutation (Fig. 1a). The mutations are spread with no significant differences in distribution in specific populations or geographical regions. Very few cases were reported from South America, and there were no case reports from Africa or Russia. The map can be used to extract country-specific information e.g., London indicated by a red arrow in Fig. 1b.

Globally, 75% of the *TGFBI* mutations reported in the over 1600 cases consisted of one of the five mutations currently detected by the available genetic test. While reports of novel *TGFBI* mutations are likely to be published, the most common *TGFBI* mutations, found at codons R124 and R555, are conversely under-reported. Therefore, it is difficult to obtain an accurate estimation of the true worldwide detection rate of *TGFBI* dystrophies within the literature.

Based on the ranking of the highest reported case numbers from our study, the effect on *TGFBI* mutation detection rates by adding six mutations to the available genetic test panel was evaluated. The reported number of cases for each of the five most common mutations and the six additional mutations proposed for the expanded test are shown in Table 1. It is noteworthy that the H626R is the fourth most prevalent mutation after R124L. This finding supports the inclusion of this mutation in an expanded panel for the diagnosis of *TGFBI* corneal dystrophy. Although only four cases of *TGFBI* corneal dystrophy associated with M502V have been reported within the literature (Supplementary Material), we discovered a heterozygous mutation for M502V in one sample. Patients with H626R and G623D TGFBI mutations (included in the six-additional mutation panel) demonstrated onset in the 4th decade or later and patients with A546D and H572R mutations presented onset during mid to late 20s, after the age at which refractive surgery may be considered. Therefore, it was included in the expanded panel.

**Fig. 2:**
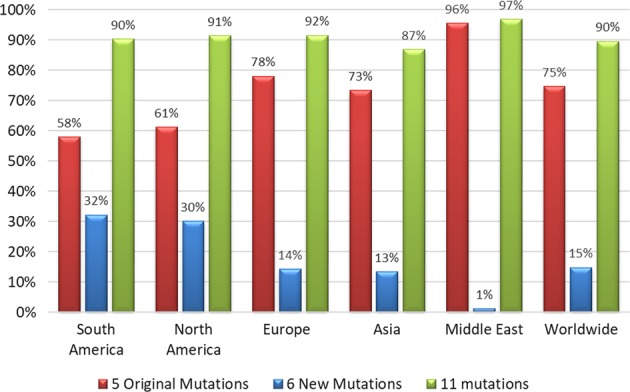
Comparison by geographic region. The original genetic test with five mutations, the six additional mutations and the proposed expanded 11 mutation panel were modeled in over 1600 reported cases. The detection rate of the available genetic test with five mutations was very close between Europe and Asia

From the cases reported in the literature, we calculated that the addition of the six new mutations to the existing panel will increase the worldwide detection rate from 75 to 90% (Fig. 2). The addition of the additional mutations to the available genetic test would theoretically increase the detection rate by 32% in South America and 30% in North America. Europe and Asia, both with a 13% increase in detection rates would also benefit from the proposed eleven mutation panel (Fig. 2).

### Global available genetic test data analysis

Since 2008, more than 600,000 samples worldwide were tested by the available genetic test; most of the samples were from Korea and Japan, where the test is used for pre-refractive surgery screening. An analysis of the global testing data demonstrated that the detection rate in Korea is approximately 15 in 10,000 people, which closely matches the reported prevalence of 1 in 870 people [11]. The detection rate of *TGFBI* mutations in Japan (3 in 10,000) was lower than that in Korea. In Korea, the test is administered as a general screening for all refractive surgery candidates, whereas in Japan, patients are first subjected to a rigorous clinical examination and only those patients who have no detected corneal abnormalities have samples submitted for the genetic test.

The clinics/hospitals in Korea and Japan use the genetic test for screening purposes as it forms part of the practice guidelines for refractive surgery. In the US, some clinics/hospitals use the test for screening during the pre-operative examination for vision corrective surgery, whereas others use it as a confirmation for clinical diagnosis or to exclude *TGFBI* mutations if the surgeon has any doubt about the imperfections noted in the patient’s cornea. European clinics utilize the test mostly for this type of clinical confirmation.

### Assessment of an expanded panel with six additional mutations

Few population studies like the 2016 UCL, Moorfield’s Corneal Dystrophy Study [5] have conducted Sanger sequencing on the entire *TGFBI* gene. This study provided us with a set of data on which to evaluate the addition of six new mutations sites to enhance the pick-up rate in a given population. In brief, the study consisted of 91 unrelated *TGFBI* corneal dystrophy cases in which 68 had a diagnosis of epithelial-stromal *TGFBI* associated dystrophy (RBCD, TBCD, LCD, and GCD) and 23 had a diagnosis of bilateral EBMD [5]. For the UK population we utilized this study as our reference, and we evaluated a set of six *TGFBI* mutations to determine whether these mutations in combination with the five mutations genetic test were appropriate. The data showed that the detection rate in the UK cohort would increase from 90 to 97% (Table 2). Other candidate mutations may be considered, such as V625D and A620D from Table 2, in order to increase the detection rate to almost 100%. This finding demonstrates that the inclusion of six additional mutations to the available genetic test, while improving the pick-up rate, will still miss some important mutations found in the UK population.

**Table 2:** Yellow highlighting indicates the theoretical results of the available genetic test

UCL/Moorfields % detection of 91 UK ethnically diverse cohort with 68 TGFBI CDs						
Clinical diagnosis	Case #	Case %	TGFBI mutation	Mutation #	Mutation %	Comments
Lattice Corneal Dystrophy	24	35%	R124C	19	28%	
			V625D	1	1%	Asian
			H626R	2	3%	
			A620D	1	1%	Asian
			G623D	1	1%	
Granular Corneal Dystrophy 1	21	31%	R555W	13	19%	
Granular Corneal Dystrophy 2			R124H	8	12%	
TB/RB CD	23	34%	R555Q	20	29%	
			R124L	1	1%	
			G623D	2	3%	
Total TGFBI CD	68		Universal test	61	90%	
			Additional 6 SNPs	5	7%	
			Total 11 SNPs	66	97%	

This test would detect 90% of the 68 *TGFBI* CD cohort identified by the Moorfield’s Corneal Dystrophy Study [5]. The green highlighting shows the six additional mutations identified through literature research. They increase the detection rate by 7%, which brings the overall detection rate in the UK to 97%.

We found that 16 of the 19 samples with clinical indications that tested negative with the original genetic test were still negative (84.2% of the total), while three tested positive (15.7% of the total) with the expanded panel. The WES results of a mother and son pair with a clinical diagnosis of late-onset of LCD were positive for a heterozygous *TGFBI* H626R mutation. Parallel real-time PCR testing showed the same heterozygous H626R mutation. The third sample was discovered to be heterozygous for M502V. The result was confirmed with Sanger sequencing Subsequent patient history revealed that the patient had very small corneal scarring on the left cornea. There was no family history of corneal dystrophy or opacity.

In accordance with evidence in the literature, we estimate that adding six mutations to the available genetic test would increase the detection rate by 15%. This coincides with the 15.7% percent increase in detection for our sample cohort (3 of 19 samples).

## Discussion

The reported prevalence of *TGFBI* corneal dystrophies in Asia is one in 870 in Korea [13] and one in 416 in China [14]. Asia has a high myopia rate, and a study conducted by Holden et al. predicted that by 2050, the Asian-Pacific population will have the highest myopia prevalence rate among all populations at 66.4% compared to the global prevalence of 49.8% [12]. With the high prevalence of myopia in these Asian populations, the use of LASIK vision correction surgery is consistently increasing and is predicted to continue to rise. With the known prevalence of *TGFBI* mutations in the Asian population and the high myopia rate, mutation testing is important in this region; subsequently, the five-mutation genetic test was initially introduced in Asian-Pacific populations.

Since the first description by Folberg et al. [15], in 1988 of *TGFBI* mutations as the cause of granular corneal dystrophy, our awareness and understanding of this disease has increased steadily. The most common R124 and R555 mutations are well documented (Supplementary Material), and additional mutations are being examined more closely to understand the next tier of common variants. In this study, we reviewed reports in the literature on various *TGFBI* corneal dystrophies to understand the prevalence of this disease. The worldwide prevalence of this disease is unknown; however, the disease outcome is debilitating. The ultimate treatment is corneal transplant, and the recurrent nature of the disease often requires subsequent corneal transplants, which is traumatic and costly to both the patients and the medical system. The preventative actions include avoid having refractive surgeries, or any eye surgeries that will injure the cornea. Special care should be taken to prevent accidental cornea injuries such as scratching or corneal trauma. Prevention and prescreening with molecular diagnostic testing to detect mutations is key.

In accordance with evidence in the literature, we determined that detection rates will improve with the addition of six mutations to the available genetic test. We did not discover geographic or population differences; therefore, the newly proposed six additional mutations are appropriate for worldwide use as an enhancement of the present genetic test. The new mutations included in an expanded test panel would considerably improve the mutation detection rate; however, this expanded test will not be able to detect all of the more than 60 *TGFBI* mutations. The law of diminishing returns, where the return of benefits fails to increase significantly with added cost must be considered, and other detection strategies may need to be evaluated, such as microarray hybridization or targeted resequencing to test for up to 70 *TGFBI* mutations referenced here and elsewhere [5].

The goal is to provide enhanced testing capability in the prescreening test prior to refractive surgery. Another objective is to close the gap between the detection rate resulting from genetic testing and clinical diagnosis. The testing of 19 samples for the presence of the six additional mutations in the expanded panel proves that the expanded genetic test will have increased detectability of *TGFBI* mutations. There are ways to achieve a 100% detection rate by using technologies such as WES. However, it is very costly and time consuming to employ WES as a screening tool. We must consider feasibility factors such as cost, turnaround time and accuracy of the refractive screening test. Ultimately, an affordable targeted sequencing panel containing all 70 TGFBI mutations as a second-tier testing should be made available, which allows patients with very rare mutations an opportunity to be tested. It is very concerning that patients carrying the rarer mutations would go through the refractive surgery without the means of being tested [16–26].

## Supplementary information

Supplementary Information
