# Supplementary material for: Evaluation of TGFBI corneal dystrophy and molecular diagnostic testing
Source: Eye (Lond). 2019 Feb 13;33(6):874–81. doi: 10.1038/s41433-019-0346-x (PMC6707296; doi:10.1038/s41433-019-0346-x)
Supplement: Supplementary file 1 — Supplementary Information [file 41433_2019_346_MOESM1_ESM.docx]

# Supplementary Material – HGMD and PubMed References

The table shows 62 *TGFBI* Variants from 120 HGMD references and 64 PubMed references. Reference papers are summarized according to *TGFBI* protein and nucleotide changes. The reported phenotype and geographical locations where the cases were discovered are also presented with each change. Comments or notes from the references are also recorded.

**Supplementary Material - 62 *TGFBI* Variants from 120 HGMD References and 64 PubMed References**

| **Protein Change** | **Nucleotide Change** | **Reported Phenotype** | **Reported Geographical Locations** | **Reference** | **Comments/Notes from Reference** |
| --- | --- | --- | --- | --- | --- |
| p.V113I | NM_000358.2:c.337G>A | Corneal dystrophy, granular | South America | [Zenteno (2006) Mol Vis 12, 331](http://www.ncbi.nlm.nih.gov/sites/entrez?cmd=Retrieve&amp;db=PubMed&amp;list_uids=16636649&amp;dopt=Abstract) | Primary literature report |
|  |  |  |  | [Weiss (2008) Cornea 27S2: S1 [Functional characterization]](http://www.ncbi.nlm.nih.gov/sites/entrez?cmd=Retrieve&amp;db=PubMed&amp;list_uids=19337156&amp;dopt=Abstract) | Variant granular corneal dystrophy. IC3D classification. |
| p.D123H | NM_000358.2:c.367G>C | Corneal dystrophy, granular | Asia | [Ha (2003) Jpn J Ophthalmol 47, 246](http://www.ncbi.nlm.nih.gov/sites/entrez?cmd=Retrieve&amp;db=PubMed&amp;list_uids=12782158&amp;dopt=Abstract) | Primary literature report |
|  |  |  |  | [Kim (2009) J Biol Chem 284: 19580 [Functional characterization]](http://www.ncbi.nlm.nih.gov/sites/entrez?cmd=Retrieve&amp;db=PubMed&amp;list_uids=19478074&amp;dopt=Abstract) |  |
|  |  |  |  | [Weiss (2008) Cornea 27S2: S1 [Functional characterization]](http://www.ncbi.nlm.nih.gov/sites/entrez?cmd=Retrieve&amp;db=PubMed&amp;list_uids=19337156&amp;dopt=Abstract) | Variant granular corneal dystrophy. IC3D classification. |
| p.R124C | NM_000358.2:c.370C>T | Corneal dystrophy, lattice type I | Asia, Europe, North America and South America | [Munier (1997) Nat Genet 15: 247 PubMed: 9054935](http://www.ncbi.nlm.nih.gov/sites/entrez?cmd=Retrieve&amp;db=PubMed&amp;list_uids=9054935&amp;dopt=Abstract) | Primary literature report |
|  |  |  |  | Blanco-Marchite (2007) Mol Vis 13:1390 PudMed: 17768377 |  |
|  |  |  |  | [Cai (2016) Genet Test Mol Biomarkers 20: 388 PubMed: 27348782](http://www.ncbi.nlm.nih.gov/sites/entrez?cmd=Retrieve&amp;db=PubMed&amp;list_uids=27348782&amp;dopt=Abstract) | Compound heterozygotes with c.1637C>A p.Ala546Asp. Phenotypic features differ from those typical of patients with single mutation. |
|  |  |  |  | [Cao (2017) Int J Ophthalmol 10: 343 PubMed: 28393022](http://www.ncbi.nlm.nih.gov/sites/entrez?cmd=Retrieve&amp;db=PubMed&amp;list_uids=20458218&amp;dopt=Abstract) |  |
|  |  |  |  | Capoluongo (2005) Eur J Ophthalmol 15(6):804 PubMed: 16329070 |  |
|  |  |  |  | Chang (2009) Arch Ophthalmol 127(5):641 PubMed: 19433713 |  |
|  |  |  |  | Chau (2003) Br J Ophthalmol 87(6):686 PubMed: 12770961 |  |
|  |  |  |  | Courtney (2015) Invest Ophthalmol 56(8):4653 PubMed: 26207300 |  |
|  |  |  |  | Dinh (1999) Ophthaomol 106(8):1490 PubMed: 10442892 |  |
|  |  |  |  | Edelstein (2010) Cornea 29: 698 PubMed: 20458218 | Avellino corneal dystrophy |
|  |  |  |  | El Kochairi (2006) Mol Vis 12: 461 PubMed: 16710170 |  |
|  |  |  |  | El-Ashry (2003) Br J Ophthalmol 87(7):839 PubMed: 12812879 |  |
|  |  |  |  | Ellies (2002) Ophthalmol 109(4):793 PubMed: 11927442 |  |
|  |  |  |  | Grothe (2013) Mol Vis 19: 593 PubMed: 23559853 |  |
|  |  |  |  | Grünauer-Kloevekorn (2005) Klin Monbl  Augenheilkd 222: 1017 PubMed: 16380889 |  |
|  |  |  |  | Han (2012) Curr Eye Res 37: 990 PubMed: 22746317 |  |
|  |  |  |  | Hao (2016) Int J Ophthalmol 9(2):198 PubMed: 26949635 |  |
|  |  |  |  | Hou (2012) Mol Vis 18: 362 PubMed: 22355247 | Flake-dot opacities with lattice lines |
|  |  |  |  | Hou (2015) Optom Vis Sci : PubMed: 25785536 | Bowman layer corneal dystrophy; occurred de novo. |
|  |  |  |  | Korvatska (1998) Am J Hum Genet 62(2):320 PubMed: 9463327 |  |
|  |  |  |  | Lisch (2014) Cornea 33: 1109 PubMed: 25055147 |  |
|  |  |  |  | Liskova (2008) Ophthalmic Res 40(2):105 PubMed: 18259096 |  |
|  |  |  |  | Liu (2008) Mol Vis 14: 1234 PubMed: 18615206 |  |
|  |  |  |  | Ma (2010) Mol Vis 16: 556 PubMed: 20360992 | Reis-Bucklers corneal dystrophy |
|  |  |  |  | Morishige (2004) Arch Ophthalmol 122: 1224 PubMed: 15302666 |  |
|  |  |  |  | Munier (2002) Invest Ophthalmol Vis Sci 43: 949 PubMed: 11923233 | Genotype-phenotype correlation. |
|  |  |  |  | Nowinska (2011) Mol Vis 17:2333 PubMed: 21921985 |  |
|  |  |  |  | Patel (2010) Cornea 29: 1215 PubMed: 20697279 | Avellino corneal dystrophy |
|  |  |  |  | Qu (2017) Biomed Rep 7(4):314 PubMed: 29085627 |  |
|  |  |  |  | Romero (2008) Mol Vis 14: 829 PubMed: 18470323 |  |
|  |  |  |  | Romero (2010) Mol Vis 16: 1601 PubMed: 20806046 |  |
|  |  |  |  | Runager (2011) J Biol Chem 286: 4951 PubMed: 21135107 | Protein stability as wild-type in vitro. |
|  |  |  |  | Schmitt–Bernard (2000) Invest Ophthalmol Vis Sci 41(6):1302 PubMed:  10798644 |  |
|  |  |  |  | Solari (2007) Eye 21(5):587 PubMed: 16440005 |  |
|  |  |  |  | Song (2017) J Cat Ref Surg 13(12):1489 PubMed: 29233738 |  |
|  |  |  |  | Wang (2017) Mol Med Rep 15: 3198 PubMed: 28358433 |  |
|  |  |  |  | Weiss (2008) Cornea 27S2: S1 PubMed: 19337156 | IC3D classification. |
|  |  |  |  | Yam (2012) Invest Ophthalmol Vis Sci 53: 5890 PubMed: 22850414 |  |
|  |  |  |  | Yang (2010) Mol Vis 16: 1186 PubMed: 20664689 |  |
|  |  |  |  | Yang (2011) Int J Ophthalmol 4(3):235 PubMed: 22553651 |  |
|  |  |  |  | Yoshida (2004) Am J Ophthalmol 137: 586 PubMed: 15013897 |  |
|  |  |  |  | Zeng (2017) Sci Rep 7(1):596 PubMed: 28377594 |  |
|  |  |  |  | Zhong (2010) Mol Vis 16: 224 PubMed: 20161820 |  |
| p.R124H | NM_000358.2:c.371G>A | Corneal dystrophy, Avellino | Asia, Europe, Middle East, North America and South America | [Munier (1997) Nat Genet 15: 247 PubMed: 9054935](http://www.ncbi.nlm.nih.gov/sites/entrez?cmd=Retrieve&amp;db=PubMed&amp;list_uids=9054935&amp;dopt=Abstract) | Primary literature report |
|  |  |  |  | [Abazi (2013) BMC Ophthalmol 13: 30 PubMed: 23837658](http://www.ncbi.nlm.nih.gov/sites/entrez?cmd=Retrieve&amp;db=PubMed&amp;list_uids=23837658&amp;dopt=Abstract) | Phenotypic variability in a family. |
|  |  |  |  | Alavi (2008) Clin Exp Ophthalmol 36(1):26 PubMed:18290950 |  |
|  |  |  |  | [Aldave (2007) Am J Ophthalmol 143: 416 PubMed: 17317389](http://www.ncbi.nlm.nih.gov/sites/entrez?cmd=Retrieve&amp;db=PubMed&amp;list_uids=21371477&amp;dopt=Abstract) | Corneal dystrophy, granular-lattice type |
|  |  |  |  | Awwad (2008) Am J Ophthalmol 145(4):656 PubMed: 18243154 |  |
|  |  |  |  | Banning (2006) Cornea 25: 482 PubMed: 16670492 |  |
|  |  |  |  | Basaiawmoit (2011) J Mol Biol 408: 503 PubMed: 21371477 | No structural differences from wild type found by small-  angle X-ray scattering modeling. |
|  |  |  |  | Bhullar (2009) Dig Ophthalmol 15(4):34 PudMed: 29270096 |  |
|  |  |  |  | Chao-Shern (2018) Eye 32(1):39 PubMed: 29192679 |  |
|  |  |  |  | Chu (2007) Arch Ophthalmol 125(5):703 PubMed: 17502515 |  |
|  |  |  |  | Cung (2004) Jon J Ophthalmol 48(1):12 PubMed: 14767644 |  |
|  |  |  |  | Davis (2011) Eyeforum |  |
|  |  |  |  | Diaper (2005) Eye (Lond) 19: 92 PubMed: 15094731 |  |
|  |  |  |  | Dogru (2001) Ophthalmol 108(4):810 PubMed: 11297503 |  |
|  |  |  |  | Du (2017) Graefes Arch Clin Exp Ophthalmol : PubMed: 28567551 | Granular corneal dystrophy; Described as c.370G>A, seq. in Figure 6. Patient also has KRT12 c.1456_1457insGAT mutation, possibly causing concomitant keratoconus. |
|  |  |  |  | El-Ashry (2003) Br J Ophthalmol 87(7):839 PubMed: 12812879 |  |
|  |  |  |  | Ellies (2002) Ophthalmol 109(4):793 PubMed: 11927442 |  |
|  |  |  |  | Ferry (1997) Trans Am Ophthalmol 95:61 PubMed: 9440163 |  |
|  |  |  |  | Folberg (1988) Ophthalmol 95(1):46 |  |
|  |  |  |  | Grünauer-Kloevekorn (2005) Klin Monbl  Augenheilkd 222: 1017 PubMed: 16380889 |  |
|  |  |  |  | Gu (2010) Mol Vis 16:1186 PMC2901189 |  |
|  |  |  |  | Han (2012) Curr Eye Res 37: 990 PubMed: 22746317 |  |
|  |  |  |  | Han (2012) Mol Vis 18: 1755 PubMed: 22815629 | Extremely varied phenotypes |
|  |  |  |  | Hao (2016) Int J Ophthalmol 9(2):198 PubMed: 26949635 |  |
|  |  |  |  | Holland (1992) Ophthalmol 99(10):1564 |  |
|  |  |  |  | Hou (2012) Mol Vis 18: 362 PubMed: 22355247 | Granular opacities |
|  |  |  |  | Huerva (2008) Eur J Ophthalmol 18(3):345 PubMed: 18465714 |  |
|  |  |  |  | Iwafuchi (2016) Case Rep Nephrol Dial 6: 106 PubMed: 27781206 | Corneal dystrophy, granular type II |
|  |  |  |  | Jun (2004) Ophthaomol 111(3):463 PubMed:15019320 |  |
|  |  |  |  | Karring (2012) Exp Eye Res 96: 163 PubMed: 22155582 |  |
|  |  |  |  | Kennedy (1996) Br J Ophthalmol 80(5):489 PubMed: 8695579 |  |
|  |  |  |  | Kim (2008) Graefes Arch Clin Exp Ophthalmol 246: 1629 PubMed: 18458933 | Phenotypic non-penetrance |
|  |  |  |  | Korvatska (1998) Am J Hum Genet 62(2):320 PubMed: 9463327 |  |
|  |  |  |  | Lee (2010) Ophthalmic Epidemiol 17(3):160 PubMed: 20455845 |  |
|  |  |  |  | Mantelli (2012) Case Rep Ophthalmol Med 2012:413010 PubMed: 22606493 |  |
|  |  |  |  | Mazzotta (2015) Case Rep Ophthalmol Med 2015: 703418 PubMed: 26221553 |  |
|  |  |  |  | Meallet (2004) Am J Ophthalmol 137(4):765 PubMed: 15059726 |  |
|  |  |  |  | Munier (2002) Invest Ophthalmol Vis Sci 43: 949 PubMed: 11923233 | Genotype-phenotype correlation. |
|  |  |  |  | Nowinska (2011) Mol Vis 17:2333 PubMed: 21921985 |  |
|  |  |  |  | Qi (2006) Zhonghua Yi Xue Yi Chuan Xue Za  Zhi 23: 310 PubMed: 16767671 |  |
|  |  |  |  | Rho (2014) Cont Lens Anterior Eye 37: 314 PubMed: 24582869 | Concomitant keratoconus and granular corneal  dystrophy type II |
|  |  |  |  | Roh (2006) Cornea 25(3):306 PubMed: 16633031 |  |
|  |  |  |  | Rossenwasser (1993) Arch Ophthalmol 111(11):1546 PubMed: 8240112 |  |
|  |  |  |  | Runager (2011) J Biol Chem 286: 4951 PubMed: 21135107 | Protein stability as wild-type in vitro. |
|  |  |  |  | Sakimoto (2015) Br J Ophthalmol 99: 26 PubMed: 25034048 |  |
|  |  |  |  | Song (2017) J Cat Ref Surg 13(12):1489 PubMed: 29233738 |  |
|  |  |  |  | Wang (2017) Mol Med Rep 15: 3198 PubMed: 28358433 |  |
|  |  |  |  | Weiss (2008) Cornea 27S2: S1 PubMed: 19337156 | Granular corneal dystrophy, type 2 (granular-lattice)  IC3D classification |
|  |  |  |  | Xie (2011) Int J Ophthalmol 4(3):275 PubMed: 22553661 |  |
|  |  |  |  | Yam (2012) Invest Ophthalmol Vis Sci 53: 5890 PubMed: 22850414 |  |
|  |  |  |  | Yamazoe (2015) PLoS One 10: e0133397 PubMed: 26197481 |  |
|  |  |  |  | Yang (2010) Mol Vis 16: 1186 PubMed: 20664689 | Granular corneal dystrophy 2 |
|  |  |  |  | Zeng (2017) Sci Rep 7(1):596 PubMed: 28377594 |  |
| p.R124L | NM_000358.2:c.371G>T | Corneal dystrophy, Reis-Buckler | Asia, Europe, North America and South America | [Okada (1998) Am J Ophthalmol 126, 535](http://www.ncbi.nlm.nih.gov/sites/entrez?cmd=Retrieve&amp;db=PubMed&amp;list_uids=9780098&amp;dopt=Abstract) | Primary literature report |
|  |  |  |  | [Cai (2016) Genet Test Mol Biomarkers 20: 388 [Additional report]](http://www.ncbi.nlm.nih.gov/sites/entrez?cmd=Retrieve&amp;db=PubMed&amp;list_uids=27348782&amp;dopt=Abstract) |  |
|  |  |  |  | Dinh (1999) Ophthaomol 106(8):1490 PubMed: 10442892 |  |
|  |  |  |  | [Evans (2016) Invest Ophthalmol Vis Sci 57: 5407 [Additional phenotype]](http://www.ncbi.nlm.nih.gov/sites/entrez?cmd=Retrieve&amp;db=PubMed&amp;list_uids=27737463&amp;dopt=Abstract) | Corneal dystrophy, Reis-Buckler/Thiel-Behnke |
|  |  |  |  | Guan (2017) Zhonghua Yi Xue Yi Chuan Xue Za Zhi 34(5):629 PubMed:  28981920 |  |
|  |  |  |  | Liang (2012) Ophthalmic Physio Opt 32(1):74 PubMed: 21899585 |  |
|  |  |  |  | Liskova (2008) Ophthalmic Res 40(2):105 PubMed: 18259096 |  |
|  |  |  |  | Munier (2002) Invest Ophthalmol Vis Sci 43: 949 PubMed: 11923233 | Genotype-phenotype correlation. |
|  |  |  |  | Paliwal (2011) Ophthalmic Res 46: 164 PubMed: 21447988 | Corneal dystrophy with amyloid and hyaline deposits |
|  |  |  |  | Qiu (2016) BMC Ophthalmol 16: 158 PubMed: 27590038 |  |
|  |  |  |  | Runager (2011) J Biol Chem 286: 4951 PubMed: 21135107 | Protein stability as wild-type in vitro. |
|  |  |  |  | Solari (2007) Eye 21(5):587 PubMed: 16440005 |  |
|  |  |  |  | Song (2017) J Cat Ref Surg 13(12):1489 PubMed: 29233738 |  |
|  |  |  |  | Tanhehco (2006) Arch Ophthalmol 124(4):589 PubMed: 16606891 |  |
|  |  |  |  | Weiss (2008) Cornea 27S2: S1 PubMed: 19337156 | IC3D classification. |
|  |  |  |  | Yam (2012) Invest Ophthalmol Vis Sci 53: 5890 PubMed: 22850414 |  |
|  |  |  |  | Zeng (2017) Sci Rep 7(1):596 PubMed: 28377594 |  |
| p.R124S | NM_000358.2:c.370C>A | Corneal dystrophy, granular type I | Asia and Europe | [Stewart (1999) Hum Mutat 14, 126](http://www.ncbi.nlm.nih.gov/sites/entrez?cmd=Retrieve&amp;db=PubMed&amp;list_uids=10425035&amp;dopt=Abstract) | Primary literature report |
|  |  |  |  | [Munier (2002) Invest Ophthalmol Vis Sci 43: 949 [Additional report]](http://www.ncbi.nlm.nih.gov/sites/entrez?cmd=Retrieve&amp;db=PubMed&amp;list_uids=11923233&amp;dopt=Abstract) | Genotype-phenotype correlation. |
|  |  |  |  | [Weiss (2008) Cornea 27S2: S1 [Functional characterisation]](http://www.ncbi.nlm.nih.gov/sites/entrez?cmd=Retrieve&amp;db=PubMed&amp;list_uids=19337156&amp;dopt=Abstract) | Variant granular corneal dystrophy. IC3D classification. |
|  |  |  |  | Yam (2012) Invest Ophthalmol Vis Sci 53: 5890 PubMed: 22850414 |  |
| p.E131D | NM_000358.2:c.393G>T | Corneal dystrophy, Schnyder | Europe | [Foja (2016) Int Ophthalmol epub, epub](http://www.ncbi.nlm.nih.gov/sites/entrez?cmd=Retrieve&amp;db=PubMed&amp;list_uids=26961680&amp;dopt=Abstract) | Primary literature report |
| p.R179* | NM_000358.2:c.535C>T | Corneal dystrophy, granular type II | Asia | [Song (2015) Ann Lab Med 35, 336](http://www.ncbi.nlm.nih.gov/sites/entrez?cmd=Retrieve&amp;db=PubMed&amp;list_uids=25932442&amp;dopt=Abstract) | Descr. as p.A179* (c.535C>T). |
| p.H403Q | NM_000358.2:c.1209T>G | Keratoconus | Europe | [Piret (2016) J Bone Miner Res 31, 1207](http://www.ncbi.nlm.nih.gov/sites/entrez?cmd=Retrieve&amp;db=PubMed&amp;list_uids=26818911&amp;dopt=Abstract) | Primary literature report |
| p.R496W | NM_000358.2:c.1486C>T | Corneal dystrophy, lattice type | Asia | [Kawasaki (2011) Br J Ophthalmol 95, 150](http://www.ncbi.nlm.nih.gov/sites/entrez?cmd=Retrieve&amp;db=PubMed&amp;list_uids=20974628&amp;dopt=Abstract) | Primary literature report |
| p.P501T | NM_000358.2:c.1501C>A | Corneal dystrophy, lattice type IIIA | Asia and Europe | [Yamamoto (1998) Am J Hum Genet 62, 719](http://www.ncbi.nlm.nih.gov/sites/entrez?cmd=Retrieve&amp;db=PubMed&amp;list_uids=9497262&amp;dopt=Abstract) | Primary literature report |
|  |  |  |  | [Chae (2016) Clin Genet 89: 678 [Additional report]](http://www.ncbi.nlm.nih.gov/sites/entrez?cmd=Retrieve&amp;db=PubMed&amp;list_uids=26748743&amp;dopt=Abstract) | Low penetrance/expressivity |
|  |  |  |  | [Ha (2000) Am J Ophthalmol 130: 119 [Additional report]](http://www.ncbi.nlm.nih.gov/sites/entrez?cmd=Retrieve&amp;db=PubMed&amp;list_uids=11004271&amp;dopt=Abstract) | low penetrance mutation. |
|  |  |  |  | Kojima (2013) Cornea 32: 1396 PubMed: 23884333 | Variant lattice corneal dystrophy, unilateral |
|  |  |  |  | Munier (2002) Invest Ophthalmol Vis Sci 43: 949 PubMed: 11923233 | Genotype-phenotype correlation. |
|  |  |  |  | Weiss (2008) Cornea 27S2: S1 PubMed: 19337156 | Variant lattice corneal dystrophy. IC3D classification. |
| p.M502V | NM_000358.2:c.1504A>G | Corneal dystrophy | Europe and South America | [Zenteno (2009) Exp Eye Res 89, 172](http://www.ncbi.nlm.nih.gov/sites/entrez?cmd=Retrieve&amp;db=PubMed&amp;list_uids=19303004&amp;dopt=Abstract) | Primary literature report |
|  |  |  |  | [Niel-Butschi (2011) Mol Vis 17: 1192 [Additional phenotype]](http://www.ncbi.nlm.nih.gov/sites/entrez?cmd=Retrieve&amp;db=PubMed&amp;list_uids=21617751&amp;dopt=Abstract) | Corneal dystrophy, Thiel-Behnke |
| p.V505D | NM_000358.2:c.1514T>A | Corneal dystrophy, lattice type I | Asia | [Tian (2005) Jpn J Ophthalmol 49, 84](http://www.ncbi.nlm.nih.gov/sites/entrez?cmd=Retrieve&amp;db=PubMed&amp;list_uids=15838722&amp;dopt=Abstract) | Primary literature report |
|  |  |  |  | [Weiss (2008) Cornea 27S2: S1 [Additional report]](http://www.ncbi.nlm.nih.gov/sites/entrez?cmd=Retrieve&amp;db=PubMed&amp;list_uids=19337156&amp;dopt=Abstract) | Variant lattice corneal dystrophy. IC3D classification. |
| p.L509R | NM_000358.2:c.1526T>G | Corneal dystrophy, epithelial basement membrane | Europe | [Boutboul (2006) Hum Mutat 27, 553](http://www.ncbi.nlm.nih.gov/sites/entrez?cmd=Retrieve&amp;db=PubMed&amp;list_uids=16652336&amp;dopt=Abstract) | Primary literature report |
|  |  |  |  | [Niel-Butschi (2011) Mol Vis 17: 1192 [Additional phenotype]](http://www.ncbi.nlm.nih.gov/sites/entrez?cmd=Retrieve&amp;db=PubMed&amp;list_uids=21617751&amp;dopt=Abstract) | Corneal dystrophy, lattice-type |
|  |  |  |  | [Weiss (2008) Cornea 27S2: S1 [Additional report]](http://www.ncbi.nlm.nih.gov/sites/entrez?cmd=Retrieve&amp;db=PubMed&amp;list_uids=19337156&amp;dopt=Abstract) | IC3D classification. |
| p.L509P | NM_000358.2:c.1526T>C | Corneal dystrophy, Reis-Buckler | Europe | [Gruenauer-Kloevekorn (2009) Br J Ophthalmol 93, 932](http://www.ncbi.nlm.nih.gov/sites/entrez?cmd=Retrieve&amp;db=PubMed&amp;list_uids=19001012&amp;dopt=Abstract) | Primary literature report |
|  |  |  |  | [Lisch (2014) Cornea 33: 1109 [Additional report]](http://www.ncbi.nlm.nih.gov/sites/entrez?cmd=Retrieve&amp;db=PubMed&amp;list_uids=25055147&amp;dopt=Abstract) |  |
|  |  |  |  | [Niel-Butschi (2011) Mol Vis 17: 1192 [Additional phenotype]](http://www.ncbi.nlm.nih.gov/sites/entrez?cmd=Retrieve&amp;db=PubMed&amp;list_uids=21617751&amp;dopt=Abstract) | Corneal dystrophy, lattice-type |
| p.R514P | NM_000358.2:c.1541G>C | Corneal dystrophy, lattice type | Asia | [Zhong (2010) Mol Vis 16, 224](http://www.ncbi.nlm.nih.gov/sites/entrez?cmd=Retrieve&amp;db=PubMed&amp;list_uids=20161820&amp;dopt=Abstract) | Primary literature report |
| p.F515L | NM_000358.2:c.1545T>A | Corneal dystrophy, lattice type | Asia | [Zhong (2010) Mol Vis 16, 224](http://www.ncbi.nlm.nih.gov/sites/entrez?cmd=Retrieve&amp;db=PubMed&amp;list_uids=20161820&amp;dopt=Abstract) | Primary literature report |
| p.S516R | NM_000358.2:c.1548C>G | Corneal dystrophy | Asia | [Paliwal (2010) Mol Vis 16, 1429](http://www.ncbi.nlm.nih.gov/sites/entrez?cmd=Retrieve&amp;db=PubMed&amp;list_uids=20680100&amp;dopt=Abstract) | Primary literature report |
| p.L518R | NM_000358.2:c.1553T>G | Corneal dystrophy, lattice intermediate  type I/IIIA | Europe | [Munier (2002) Invest Ophthalmol Vis Sci 43, 949](http://www.ncbi.nlm.nih.gov/sites/entrez?cmd=Retrieve&amp;db=PubMed&amp;list_uids=11923233&amp;dopt=Abstract) | Primary literature report |
| p.L518P | NM_000358.2:c.1553T>C | Corneal dystrophy, lattice intermediate type I/IIIA | Europe, Asia | [Endo (1999) Am J Ophthalmol 128, 104](http://www.ncbi.nlm.nih.gov/sites/entrez?cmd=Retrieve&amp;db=PubMed&amp;list_uids=10482106&amp;dopt=Abstract) | Primary literature report |
|  |  |  |  | [Munier (2002) Invest Ophthalmol Vis Sci 43: 949 [Additional report]](http://www.ncbi.nlm.nih.gov/sites/entrez?cmd=Retrieve&amp;db=PubMed&amp;list_uids=11923233&amp;dopt=Abstract) | Genotype-phenotype correlation. |
|  |  |  |  | [Weiss (2008) Cornea 27S2: S1 [Additional report]](http://www.ncbi.nlm.nih.gov/sites/entrez?cmd=Retrieve&amp;db=PubMed&amp;list_uids=19337156&amp;dopt=Abstract) | Variant lattice corneal dystrophy. IC3D classification. |
| p.I522N | NM_000358.2:c.1565T>A | Corneal dystrophy, lattice type I | Asia | [Zhang (2009) Mol Vis 15, 2498](http://www.ncbi.nlm.nih.gov/sites/entrez?cmd=Retrieve&amp;db=PubMed&amp;list_uids=19956413&amp;dopt=Abstract) | Primary literature report |
| p.L527R | NM_000358.2:c.1580T>G | Corneal dystrophy, lattice type, with deep deposits | Asia and Europe | [Fujiki (1998) Hum Genet 103: 286 PubMed: 9799082](http://www.ncbi.nlm.nih.gov/sites/entrez?cmd=Retrieve&amp;db=PubMed&amp;list_uids=9799082&amp;dopt=Abstract) | Primary literature report |
|  |  |  |  | [Funayama (2006) Jpn J Ophthalmol 50: 62 PubMed: 16453189](http://www.ncbi.nlm.nih.gov/sites/entrez?cmd=Retrieve&amp;db=PubMed&amp;list_uids=11413411&amp;dopt=Abstract) | Corneal dystrophy, lattice type III |
|  |  |  |  | Hirano (2001) Cornea 20: 525 PubMed: 11413411 |  |
|  |  |  |  | Kawashima (2005) Nippon Ganka Gakkai Zasshi 109: 93 PubMed: 15770959 |  |
|  |  |  |  | Kim (2014) Korean J Ophthalmol 28: 83 PubMed: 24505203 | Corneal dystrophy, lattice type IV |
|  |  |  |  | [Munier (2002) Invest Ophthalmol Vis Sci 43: 949 PubMed: 11923233](http://www.ncbi.nlm.nih.gov/sites/entrez?cmd=Retrieve&amp;db=PubMed&amp;list_uids=24505203&amp;dopt=Abstract) | Genotype-phenotype correlation. |
|  |  |  |  | Ohnishi (2010) Jpn J Ophthalmol 54: 628 PubMed: 21191728 | Corneal dystrophy, lattice type |
|  |  |  |  | Weiss (2008) Cornea 27S2: S1 PubMed: 19337156 | Variant lattice corneal dystrophy. IC3D classification. |
|  |  |  |  | Yamada (2005) Br J Ophthalmol 89: 771 PubMed: 15923518 |  |
| p.G535* | NM_000358.2:c.1603G>T | Keratoconus | Asia | [Guan (2011) Zhonghua Yi Xue Yi Chuan Xue Za Zhi 28, 152](http://www.ncbi.nlm.nih.gov/sites/entrez?cmd=Retrieve&amp;db=PubMed&amp;list_uids=21462125&amp;dopt=Abstract) | Primary literature report |
|  |  |  |  | [Guan (2012) Gene 503: 137 [Additional report]](http://www.ncbi.nlm.nih.gov/sites/entrez?cmd=Retrieve&amp;db=PubMed&amp;list_uids=22575726&amp;dopt=Abstract) |  |
|  |  |  |  | [Xiong (2015) Science 347: 1254806 [Additional report]](http://www.ncbi.nlm.nih.gov/sites/entrez?cmd=Retrieve&amp;db=PubMed&amp;list_uids=25525159&amp;dopt=Abstract) | predicted to induce a large splicing change - Table S4. |
| p.T538R | NM_000358.2:c.1613C>G | Corneal dystrophy, lattice intermediate type I/IIIA | Europe | [Munier (2002) Invest Ophthalmol Vis Sci 43, 949](http://www.ncbi.nlm.nih.gov/sites/entrez?cmd=Retrieve&amp;db=PubMed&amp;list_uids=11923233&amp;dopt=Abstract) | Primary literature report |
|  |  |  |  | [Weiss (2008) Cornea 27S2: S1 [Additional report]](http://www.ncbi.nlm.nih.gov/sites/entrez?cmd=Retrieve&amp;db=PubMed&amp;list_uids=19337156&amp;dopt=Abstract) | Variant lattice corneal dystrophy. IC3D classification. |
| p.T538P | NM_000358.2:c.1612A>C | Corneal dystrophy, lattice type | Asia | [Yu (2006) J Genet 85, 73](http://www.ncbi.nlm.nih.gov/sites/entrez?cmd=Retrieve&amp;db=PubMed&amp;list_uids=16809844&amp;dopt=Abstract) | Primary literature report |
|  |  |  |  | [Weiss (2008) Cornea 27S2: S1 [Additional report]](http://www.ncbi.nlm.nih.gov/sites/entrez?cmd=Retrieve&amp;db=PubMed&amp;list_uids=19337156&amp;dopt=Abstract) | Variant lattice corneal dystrophy. IC3D classification. |
|  |  |  |  | [Zhu (2012) Mol Vis 18: 1156 [Functional characterisation]](http://www.ncbi.nlm.nih.gov/sites/entrez?cmd=Retrieve&amp;db=PubMed&amp;list_uids=22605926&amp;dopt=Abstract) |  |
| p.V539D | NM_000358.2:c.1616T>A | Corneal dystrophy, lattice type | Asia | [Chakravarthi (2005) Invest Ophthalmol Vis Sci 46, 121](http://www.ncbi.nlm.nih.gov/sites/entrez?cmd=Retrieve&amp;db=PubMed&amp;list_uids=15623763&amp;dopt=Abstract) | Primary literature report |
|  |  |  |  | [Weiss (2008) Cornea 27S2: S1 [Additional report]](http://www.ncbi.nlm.nih.gov/sites/entrez?cmd=Retrieve&amp;db=PubMed&amp;list_uids=19337156&amp;dopt=Abstract) | Variant lattice corneal dystrophy. IC3D classification. |
| p.F540S | NM_000358.2:c.1619T>C | Corneal dystrophy, lattice type | Europe | [Stix (2005) Invest Ophthalmol Vis Sci 46, 1133](http://www.ncbi.nlm.nih.gov/sites/entrez?cmd=Retrieve&amp;db=PubMed&amp;list_uids=15790870&amp;dopt=Abstract) | Primary literature report |
|  |  |  |  | [Weiss (2008) Cornea 27S2: S1 [Additional report]](http://www.ncbi.nlm.nih.gov/sites/entrez?cmd=Retrieve&amp;db=PubMed&amp;list_uids=19337156&amp;dopt=Abstract) | Variant lattice corneal dystrophy. IC3D classification. |
| p.P542R | NM_000358.2:c.1625C>G | Corneal dystrophy, lattice type | Asia | [Cho (2012) Mol Vis 18, 2012](http://www.ncbi.nlm.nih.gov/sites/entrez?cmd=Retrieve&amp;db=PubMed&amp;list_uids=22876129&amp;dopt=Abstract) | Primary literature report |
| p.N544S | NM_000358.2:c.1631A>G | Corneal dystrophy, lattice intermediate type I/IIIA | Asia and Europe | [Mashima (2000) Am J Ophthalmol 130: 516 PubMed: 11024425](http://www.ncbi.nlm.nih.gov/entrez/query.fcgi?cmd=Retrieve&amp;db=PubMed&amp;list_uids=11024425&amp;dopt=Abstract) | Primary literature report |
|  |  |  |  | [Kawashima (2005) Nippon Ganka Gakkai Zasshi 109: 93 PubMed: 15770959](http://www.ncbi.nlm.nih.gov/sites/entrez?cmd=Retrieve&amp;db=PubMed&amp;list_uids=15770959&amp;dopt=Abstract) |  |
|  |  |  |  | [Munier (2002) Invest Ophthalmol Vis Sci 43: 949 PubMed: 11923233](http://www.ncbi.nlm.nih.gov/sites/entrez?cmd=Retrieve&amp;db=PubMed&amp;list_uids=11923233&amp;dopt=Abstract) | Genotype-phenotype correlation. |
|  |  |  |  | [Nakagawa Asahina (2004) Nippon Ganka Gakkai](http://www.ncbi.nlm.nih.gov/sites/entrez?cmd=Retrieve&amp;db=PubMed&amp;list_uids=15559315&amp;dopt=Abstract)  [Zasshi 108: 618 PubMed: 15559315](http://www.ncbi.nlm.nih.gov/sites/entrez?cmd=Retrieve&amp;db=PubMed&amp;list_uids=15559315&amp;dopt=Abstract) |  |
|  |  |  |  | [Weiss (2008) Cornea 27S2: S1 PubMed: 19337156](http://www.ncbi.nlm.nih.gov/sites/entrez?cmd=Retrieve&amp;db=PubMed&amp;list_uids=19337156&amp;dopt=Abstract) | Variant lattice corneal dystrophy. IC3D classification. |
| p.A546D | NM_000358.2:c.1637C>A | Corneal dystrophy, lattice type | Asia, Europe, North America and South America | [Aldave (2004) Am J Ophthalmol 138: 772 PubMed: 15531312](http://www.ncbi.nlm.nih.gov/sites/entrez?cmd=Retrieve&amp;db=PubMed&amp;list_uids=15531312&amp;dopt=Abstract) | Primary literature report |
|  |  |  |  | [Aldave (2004) Ophthalmology 111: 1407 PubMed: 15234146](http://www.ncbi.nlm.nih.gov/sites/entrez?cmd=Retrieve&amp;db=PubMed&amp;list_uids=15234146&amp;dopt=Abstract) | Anterior corneal stromal opacification |
|  |  |  |  | [Cao (2017) Int J Ophthalmol 10: 343 PubMed: 28393022](http://www.ncbi.nlm.nih.gov/sites/entrez?cmd=Retrieve&amp;db=PubMed&amp;list_uids=22355247&amp;dopt=Abstract) | Compound heterozygotes with c.370C>T p.Arg124Cys. Phenotypic features differ from those typical of patients  with single mutation. |
|  |  |  |  | Correa-Gomez (2007) Mol Vis 13: 1695 PubMed: 17893671 |  |
|  |  |  |  | [Eifrig (2004) Ophthaomol 111(6):1108 PubMed: 15177960](https://www.ncbi.nlm.nih.gov/pubmed/15177960) |  |
|  |  |  |  | Hou (2012) Mol Vis 18: 362 PubMed: 22355247 | Polymorphic dots with lattice lines |
|  |  |  |  | [Klintworth (2004) Invest Ophthalmol Vis Sci 45(5):1382 PubMed: 15111592](https://www.ncbi.nlm.nih.gov/pubmed/?term=Two%2BMutations%2Bin%2Bthe%2BTGFBI%2B(BIGH3)%2BGene%2BAssociated%2Bwith%2BLattice%2BCorneal%2BDystrophy%2Bin%2Ban%2BExtensively%2BStudied%2BFamily) |  |
|  |  |  |  | Long (2011) J Zhejiang Univ Sci B 12: 287 PubMed: 21462384 | Corneal dystrophy, granular |
|  |  |  |  | Poulsen (2014) Proteomics Clin Appl 8: 168 PubMed: 24302499 |  |
|  |  |  |  | Weiss (2008) Cornea 27S2: S1 PubMed: 19337156 | Variant lattice corneal dystrophy. IC3D classification. |
| p.A546T | NM_000358.2:c.1636G>A | Corneal dystrophy, lattice type IIIA | Europe | [Dighiero (2000) Am J Ophthalmol 129, 248](http://www.ncbi.nlm.nih.gov/sites/entrez?cmd=Retrieve&amp;db=PubMed&amp;list_uids=10682981&amp;dopt=Abstract) | Primary literature report |
|  |  |  |  | [Koldsø (2015) Biochemistry 54: 5546 [Additional report]](http://www.ncbi.nlm.nih.gov/sites/entrez?cmd=Retrieve&amp;db=PubMed&amp;list_uids=26305369&amp;dopt=Abstract) | Molecular dynamics simulations and principal  component analysis. |
|  |  |  |  | [Munier (2002) Invest Ophthalmol Vis Sci 43: 949 [Additional report]](http://www.ncbi.nlm.nih.gov/sites/entrez?cmd=Retrieve&amp;db=PubMed&amp;list_uids=11923233&amp;dopt=Abstract) | Genotype-phenotype correlation. |
|  |  |  |  | Runager (2011) J Biol Chem 286: 4951 PubMed: 21135107 | Reduced protein stability compared to wild-type in  vitro. |
|  |  |  |  | Weiss (2008) Cornea 27S2: S1 PubMed: 19337156 | Variant lattice corneal dystrophy. IC3D classification. |
| p.F547C | NM_000358.2:c.1640T>G | Corneal dystrophy, granular | Europe | [Foja (2016) Int Ophthalmol epub, epub](http://www.ncbi.nlm.nih.gov/sites/entrez?cmd=Retrieve&amp;db=PubMed&amp;list_uids=26961680&amp;dopt=Abstract) | Primary literature report |
| p.F547S | NM_000358.2:c.1640T>C | Corneal dystrophy, lattice type | Europe | [Takacs (2007) Mol Vis 13, 1976](http://www.ncbi.nlm.nih.gov/sites/entrez?cmd=Retrieve&amp;db=PubMed&amp;list_uids=17982422&amp;dopt=Abstract) | Primary literature report |
|  |  |  |  | [Weiss (2008) Cornea 27S2: S1 [Additional report]](http://www.ncbi.nlm.nih.gov/sites/entrez?cmd=Retrieve&amp;db=PubMed&amp;list_uids=19337156&amp;dopt=Abstract) | Variant lattice corneal dystrophy. IC3D classification. |
| p.R548P | NM_000358.2:c.1643G>C | Corneal dystrophy, lattice type | Asia | [Chae (2016) Clin Genet 89, 678](http://www.ncbi.nlm.nih.gov/sites/entrez?cmd=Retrieve&amp;db=PubMed&amp;list_uids=26748743&amp;dopt=Abstract) | Primary literature report |
| p.L550P | NM_000358.2:c.1649T>C | Corneal dystrophy, granular | Asia and South America | [Zenteno (2009) Exp Eye Res 89, 172](http://www.ncbi.nlm.nih.gov/sites/entrez?cmd=Retrieve&amp;db=PubMed&amp;list_uids=19303004&amp;dopt=Abstract) | Primary literature report |
|  |  |  |  | [Lakshminarayanan (2011) Br J Ophthalmol 95: 1457 [Additional phenotype]](http://www.ncbi.nlm.nih.gov/sites/entrez?cmd=Retrieve&amp;db=PubMed&amp;list_uids=21835759&amp;dopt=Abstract) | Corneal dystrophy, Avellino |
| p.P551Q | NM_000358.2:c.1652C>A | Corneal dystrophy, lattice type | Europe and North America | [Aldave (2004) Am J Ophthalmol 138, 772](http://www.ncbi.nlm.nih.gov/sites/entrez?cmd=Retrieve&amp;db=PubMed&amp;list_uids=15531312&amp;dopt=Abstract) | Primary literature report |
|  |  |  |  | [Aldave (2004) Ophthalmology 111: 1407 [Additional phenotype]](http://www.ncbi.nlm.nih.gov/sites/entrez?cmd=Retrieve&amp;db=PubMed&amp;list_uids=15234146&amp;dopt=Abstract) | Anterior corneal stromal opacification |
|  |  |  |  | [Poulsen (2014) Proteomics Clin Appl 8: 168 [Functional characterisation]](http://www.ncbi.nlm.nih.gov/sites/entrez?cmd=Retrieve&amp;db=PubMed&amp;list_uids=24302499&amp;dopt=Abstract) | A546D/P551Q double mutant has different properties  to A546D mutant alone. |
|  |  |  |  | [Weiss (2008) Cornea 27S2: S1 PubMed: 19337156](http://www.ncbi.nlm.nih.gov/sites/entrez?cmd=Retrieve&amp;db=PubMed&amp;list_uids=19337156&amp;dopt=Abstract) | Variant lattice corneal dystrophy. IC3D classification. |
| p.R555Q | NM_000358.2:c.1664G>A | Corneal dystrophy, Thiel-Behnke | Asia, Europe, North America and South America | [Munier (1997) Nat Genet 15: 247 PubMed: 9054935](http://www.ncbi.nlm.nih.gov/sites/entrez?cmd=Retrieve&amp;db=PubMed&amp;list_uids=9054935&amp;dopt=Abstract) | Primary literature report. Phenotype descr. as Reis-  Bucklers Corneal Dystrophy. |
|  |  |  |  | Gear (2005) Br J Ophthalmol 89(4):518 PubMed: 15774937 |  |
|  |  |  |  | Hao (2016) Int J Ophthalmol 9(2):198 PubMed: 26949635 |  |
|  |  |  |  | [Hou (2012) Mol Vis 18: 362 PubMed: 22355247](http://www.ncbi.nlm.nih.gov/sites/entrez?cmd=Retrieve&amp;db=PubMed&amp;list_uids=22355247&amp;dopt=Abstract) | Reticular superficial opacities |
|  |  |  |  | [Kobayashi (2007) Ophthalmology 114: 69 PubMed: 17198850](http://www.ncbi.nlm.nih.gov/sites/entrez?cmd=Retrieve&amp;db=PubMed&amp;list_uids=26305369&amp;dopt=Abstract) |  |
|  |  |  |  | Koldsø (2015) Biochemistry 54: 5546 PubMed: 26305369 | Molecular dynamics simulations and principal  component analysis. |
|  |  |  |  | Korvatska (1998) Am J Hum Genet 62(2):320 PubMed: 9463327 |  |
|  |  |  |  | Kuchle (1995) Cornea 14(4):333 PubMed: 7671605 |  |
|  |  |  |  | Liskova (2008) Ophthalmic Res 40(2):105 PubMed: 18259096 |  |
|  |  |  |  | Munier (2002) Invest Ophthalmol Vis Sci 43: 949 PubMed: 11923233 | Genotype-phenotype correlation. |
|  |  |  |  | Nowinska (2011) Mol Vis 17:2333 PubMed: 21921985 |  |
|  |  |  |  | Piao (2012) J Int Med Res 40: 1149 PubMed: 22906289 | Corneal dystrophy, Reis-Buckler |
|  |  |  |  | Qi (2006) Zhonghua Yi Xue Yi Chuan Xue Za  Zhi 23: 310 PubMed: 16767671 | Thiel-Behnke corneal dystrophy |
|  |  |  |  | Qin (2010) Zhonghua Yi Xue Yi Chuan Xue Za Zhi 27(5):489 PubMed:  20931522 |  |
|  |  |  |  | Runager (2011) J Biol Chem 286: 4951 PubMed: 21135107 | Reduced protein stability compared to wild-type in  vitro. |
|  |  |  |  | Solari (2007) Eye 21(5):587 PubMed: 16440005 |  |
|  |  |  |  | Song (2017) J Cat Ref Surg 13(12):1489 PubMed: 29233738 |  |
|  |  |  |  | Weiss (2008) Cornea 27S2: S1 PubMed: 19337156 | IC3D classification. |
|  |  |  |  | Zeng (2017) Sci Rep 7(1):596 PubMed: 28377594 |  |
| p.R555W | NM_000358.2:c.1663C>T | Corneal dystrophy, granular type I | Asia, Europe, New Zealand, North America and South America | [Munier (1997) Nat Genet 15: 247 PubMed: 9054935](http://www.ncbi.nlm.nih.gov/sites/entrez?cmd=Retrieve&amp;db=PubMed&amp;list_uids=9054935&amp;dopt=Abstract) | Primary literature report |
|  |  |  |  | Blanco-Marchite (2007) Mol Vis 13:1390 PudMed: 17768377 |  |
|  |  |  |  | Courtney (2015) Invest Ophthalmol 56(8):4653 PubMed: 26207300 |  |
|  |  |  |  | Cung (2004) Jon J Ophthalmol 48(1):12 PubMed: 14767644 |  |
|  |  |  |  | [Elavazhagan (2012) Protein Expr Purif 84: 108 PubMed: 22575305](http://www.ncbi.nlm.nih.gov/sites/entrez?cmd=Retrieve&amp;db=PubMed&amp;list_uids=22575305&amp;dopt=Abstract) | Solubility and secondary structure as wild-type.  Increased stability comp. to wild-type. |
|  |  |  |  | Ellies (2002) Ophthalmol 109(4):793 PubMed: 11927442 |  |
|  |  |  |  | Frising (2006) Cornea 25(5):614 PubMed: 16783153 |  |
|  |  |  |  | Garg (2010) Indian J Ophthalmol 58(4):328 PubMed: 20534926 |  |
|  |  |  |  | [Grothe (2013) Mol Vis 19: 593 PubMed: 23559853](http://www.ncbi.nlm.nih.gov/sites/entrez?cmd=Retrieve&amp;db=PubMed&amp;list_uids=23559853&amp;dopt=Abstract) |  |
|  |  |  |  | [Han (2012) Curr Eye Res 37: 990 PubMed: 22746317](http://www.ncbi.nlm.nih.gov/sites/entrez?cmd=Retrieve&amp;db=PubMed&amp;list_uids=22746317&amp;dopt=Abstract) |  |
|  |  |  |  | Hao (2016) Int J Ophthalmol 9(2):198 PubMed: 26949635 |  |
|  |  |  |  | [Hou (2003) J Formos Med Assoc 102: 117 PubMed: 12709742](http://www.ncbi.nlm.nih.gov/sites/entrez?cmd=Retrieve&amp;db=PubMed&amp;list_uids=12709742&amp;dopt=Abstract) |  |
|  |  |  |  | [Hou (2012) Mol Vis 18: 362 PubMed: 22355247](http://www.ncbi.nlm.nih.gov/sites/entrez?cmd=Retrieve&amp;db=PubMed&amp;list_uids=22355247&amp;dopt=Abstract) | Bread crumb opactities |
|  |  |  |  | Kaluzny (2008) Cornea 27(7):830 PubMed: 18650671 |  |
|  |  |  |  | [Kaluzny (2008) Cornea 27: 830 PubMed: 18650671](http://www.ncbi.nlm.nih.gov/sites/entrez?cmd=Retrieve&amp;db=PubMed&amp;list_uids=26305369&amp;dopt=Abstract) |  |
|  |  |  |  | [Kannabiran (2005) Arch Ophthalmol 123: 1127 PubMed: 16087849](http://www.ncbi.nlm.nih.gov/sites/entrez?cmd=Retrieve&amp;db=PubMed&amp;list_uids=11923233&amp;dopt=Abstract) |  |
|  |  |  |  | Kattan (2017) Cornea 36(2):210 PubMed: 28060069 |  |
|  |  |  |  | Kocak-Altintas (2001) Cornea 20(1):64 PubMed: 11189007 |  |
|  |  |  |  | [Koldsø (2015) Biochemistry 54: 5546 PubMed: 26305369](http://www.ncbi.nlm.nih.gov/sites/entrez?cmd=Retrieve&amp;db=PubMed&amp;list_uids=16767671&amp;dopt=Abstract) | Molecular dynamics simulations and principal  component analysis. |
|  |  |  |  | Korvatska (1998) Am J Hum Genet 62(2):320 PubMed: 9463327 |  |
|  |  |  |  | Lei (2009) Chin Med J 122(22):2691 PubMed: 19951597 |  |
|  |  |  |  | Liskova (2008) Ophthalmic Res 40(2):105 PubMed: 18259096 |  |
|  |  |  |  | Moller (1990) Acta Ophthalmol 68(1):97 PubMed: 2336942 |  |
|  |  |  |  | Moller (1990) Acta Ophthalmol 68(3):297 PubMed: 2392905 |  |
|  |  |  |  | [Munier (2002) Invest Ophthalmol Vis Sci 43: 949 PubMed: 11923233](http://www.ncbi.nlm.nih.gov/sites/entrez?cmd=Retrieve&amp;db=PubMed&amp;list_uids=23828476&amp;dopt=Abstract) | Genotype-phenotype correlation. |
|  |  |  |  | Nowinska (2011) Mol Vis 17:2333 PubMed: 21921985 |  |
|  |  |  |  | [Qi (2006) Zhonghua Yi Xue Yi Chuan Xue Za](http://www.ncbi.nlm.nih.gov/sites/entrez?cmd=Retrieve&amp;db=PubMed&amp;list_uids=21836353&amp;dopt=Abstract)  [Zhi 23: 310 PubMed: 16767671](http://www.ncbi.nlm.nih.gov/sites/entrez?cmd=Retrieve&amp;db=PubMed&amp;list_uids=21836353&amp;dopt=Abstract) | Granular corneal dystrophy |
|  |  |  |  | [Rama (2013) Arq Bras Oftalmol 76: 126 PubMed: 23828476](http://www.ncbi.nlm.nih.gov/sites/entrez?cmd=Retrieve&amp;db=PubMed&amp;list_uids=21135107&amp;dopt=Abstract) |  |
|  |  |  |  | [Rathi (2011) Indian J Ophthalmol 59: 398 PubMed: 21836353](http://www.ncbi.nlm.nih.gov/sites/entrez?cmd=Retrieve&amp;db=PubMed&amp;list_uids=24129074&amp;dopt=Abstract) | keratoconus with granular dystrophy |
|  |  |  |  | [Runager (2011) J Biol Chem 286: 4951 PubMed: 21135107](http://www.ncbi.nlm.nih.gov/sites/entrez?cmd=Retrieve&amp;db=PubMed&amp;list_uids=19948560&amp;dopt=Abstract) | Increased protein stability compared to wild-type in  vitro. |
|  |  |  |  | Solari (2007) Eye 21(5):587 PubMed: 16440005 |  |
|  |  |  |  | Song (2017) J Cat Ref Surg 13(12):1489 PubMed: 29233738 |  |
|  |  |  |  | [Underhaug (2013) Biochim Biophys Acta 1834: 2812 PubMed: 24129074](http://www.ncbi.nlm.nih.gov/sites/entrez?cmd=Retrieve&amp;db=PubMed&amp;list_uids=19337156&amp;dopt=Abstract) |  |
|  |  |  |  | [Vincent (2010) Br J Ophthalmol 94: 836 PubMed: 19948560](http://www.ncbi.nlm.nih.gov/sites/entrez?cmd=Retrieve&amp;db=PubMed&amp;list_uids=22850414&amp;dopt=Abstract) | Corneal dystrophy, granular |
|  |  |  |  | [Weiss (2008) Cornea 27S2: S1 PubMed: 19337156](http://www.ncbi.nlm.nih.gov/sites/entrez?cmd=Retrieve&amp;db=PubMed&amp;list_uids=20664689&amp;dopt=Abstract) | Granular corneal dystrophy, type 1 (classic). IC3D  classification. |
|  |  |  |  | Yam (2012) Invest Ophthalmol Vis Sci 53: 5890 PubMed: 22850414 |  |
|  |  |  |  | Yang (2010) Mol Vis 16: 1186 PubMed: 20664689 | Granular corneal dystrophy 1 |
|  |  |  |  | Yu (2015) BMC Ophthalmol 15: 131 PubMed: 26464103 | Corneal dystrophy, Thiel-Behnke |
|  |  |  |  | Zeng (2017) Sci Rep 7(1):596 PubMed: 28377594 |  |
|  |  |  |  | Zhao (2013) Int J Ophthalmol 6: 458 PubMed: 23991378 | Granular corneal dystrophy type I, atypical |
|  |  |  |  | [Zhu (2011) Mol Vis 17: 225 PubMed: 21264234](http://www.ncbi.nlm.nih.gov/sites/entrez?cmd=Retrieve&amp;db=PubMed&amp;list_uids=26464103&amp;dopt=Abstract) | Granular corneal dystrophy 1, new type |
|  |  |  |  | [Zhu (2012) Mol Vis 18: 1156 PubMed: 22605926](http://www.ncbi.nlm.nih.gov/sites/entrez?cmd=Retrieve&amp;db=PubMed&amp;list_uids=23991378&amp;dopt=Abstract) |  |
| p.L558R | NM_000358.2:c.1673T>G | Corneal dystrophy, lattice type | Europe | [Dudakova (2016) Ophthalmic Genet 37, 473](http://www.ncbi.nlm.nih.gov/sites/entrez?cmd=Retrieve&amp;db=PubMed&amp;list_uids=27028151&amp;dopt=Abstract) | Primary literature report |
| p.L558P | NM_000358.2:c.1673T>C | Corneal dystrophy, lattice type | Europe | [Livshits (2008) Hum Genet 124 296](http://link.springer.com/journal/439) | Primary literature report |
|  |  |  |  | [Pampukha (2009) Ophthalmologica 223: 207 [Additional report]](http://www.ncbi.nlm.nih.gov/sites/entrez?cmd=Retrieve&amp;db=PubMed&amp;list_uids=19221447&amp;dopt=Abstract) |  |
| p.L559V | NM_000358.2:c.1675T>G | Corneal dystrophy | Asia | [Paliwal (2010) Mol Vis 16, 1429](http://www.ncbi.nlm.nih.gov/sites/entrez?cmd=Retrieve&amp;db=PubMed&amp;list_uids=20680100&amp;dopt=Abstract) | Primary literature report |
| p.L565P | NM_000358.2:c.1694T>C | Corneal dystrophy, lattice type | Europe | [Ołdak (2014) Cornea 33, 294](http://www.ncbi.nlm.nih.gov/sites/entrez?cmd=Retrieve&amp;db=PubMed&amp;list_uids=24473223&amp;dopt=Abstract) | Primary literature report |
| p.L569R | NM_000358.2:c.1706T>G | Corneal dystrophy, lattice type I | North America | [Warren (2003) Am J Ophthalmol 136, 872](http://www.ncbi.nlm.nih.gov/sites/entrez?cmd=Retrieve&amp;db=PubMed&amp;list_uids=14597039&amp;dopt=Abstract) | Primary literature report |
|  |  |  |  | [Weiss (2008) Cornea 27S2: S1 [Additional report]](http://www.ncbi.nlm.nih.gov/sites/entrez?cmd=Retrieve&amp;db=PubMed&amp;list_uids=19337156&amp;dopt=Abstract) | Variant lattice corneal dystrophy. IC3D classification. |
| p.L569Q | NM_000358.2:c.1706T>A | Corneal dystrophy, lattice type I | Asia | [Song (2015) Ann Lab Med 35, 336](http://www.ncbi.nlm.nih.gov/sites/entrez?cmd=Retrieve&amp;db=PubMed&amp;list_uids=25932442&amp;dopt=Abstract) | Primary literature report |
| p.H572R | NM_000358.2:c.1715A>G | Corneal dystrophy, lattice type I | Asia and South America | [Atchaneeyasakul (2006) Jpn J Ophthalmol 50, 403](http://www.ncbi.nlm.nih.gov/sites/entrez?cmd=Retrieve&amp;db=PubMed&amp;list_uids=17013691&amp;dopt=Abstract) | Primary literature report |
|  |  |  |  | [Elavazhagan (2012) Protein Expr Purif 84: 108 [Functional characterisation]](http://www.ncbi.nlm.nih.gov/sites/entrez?cmd=Retrieve&amp;db=PubMed&amp;list_uids=22575305&amp;dopt=Abstract) | Solubility and secondary structure as wild-type.  Increased stability comp. to wild-type. |
|  |  |  |  | [Romero (2010) Mol Vis 16: 1601 [Additional report]](http://www.ncbi.nlm.nih.gov/sites/entrez?cmd=Retrieve&amp;db=PubMed&amp;list_uids=20806046&amp;dopt=Abstract) |  |
|  |  |  |  | [Weiss (2008) Cornea 27S2: S1 PubMed: 19337156](http://www.ncbi.nlm.nih.gov/sites/entrez?cmd=Retrieve&amp;db=PubMed&amp;list_uids=19337156&amp;dopt=Abstract) | Variant lattice corneal dystrophy. IC3D classification. |
|  |  |  |  | [Zhong (2010) Mol Vis 16: 224 PubMed: 20161820](http://www.ncbi.nlm.nih.gov/sites/entrez?cmd=Retrieve&amp;db=PubMed&amp;list_uids=20161820&amp;dopt=Abstract) |  |
| p.G594V | NM_000358.2:c.1781G>T | Corneal dystrophy, lattice type | Asia | [Chakravarthi (2005) Invest Ophthalmol Vis Sci 46, 121](http://www.ncbi.nlm.nih.gov/sites/entrez?cmd=Retrieve&amp;db=PubMed&amp;list_uids=15623763&amp;dopt=Abstract) | Primary literature report |
|  |  |  |  | [Weiss (2008) Cornea 27S2: S1 [Additional report]](http://www.ncbi.nlm.nih.gov/sites/entrez?cmd=Retrieve&amp;db=PubMed&amp;list_uids=19337156&amp;dopt=Abstract) | Variant lattice corneal dystrophy. IC3D classification. |
| p.V613G | NM_000358.2:c.1838T>G | Corneal dystrophy, lattice type | Europe | [Niel-Butschi (2011) Mol Vis 17, 1192](http://www.ncbi.nlm.nih.gov/sites/entrez?cmd=Retrieve&amp;db=PubMed&amp;list_uids=21617751&amp;dopt=Abstract) | Primary literature report |
| p.M619K | NM_000358.2:c.1856T>A | Corneal dystrophy, combined granular- lattice type, variant of | North America and South America | [Aldave (2008) Arch Ophthalmol 126: 371 PubMed: 18332318](http://www.ncbi.nlm.nih.gov/sites/entrez?cmd=Retrieve&amp;db=PubMed&amp;list_uids=18332318&amp;dopt=Abstract) | Primary literature report |
|  |  |  |  | Anandalakshmi (2017) Biochem J 474: 1705 PubMed: 28381645 |  |
|  |  |  |  | [Gonzalez-Rodriguez (2014) Graefes Arch Clin Exp](http://www.ncbi.nlm.nih.gov/sites/entrez?cmd=Retrieve&amp;db=PubMed&amp;list_uids=24801599&amp;dopt=Abstract)  [Ophthalmol : PubMed: 24801599](http://www.ncbi.nlm.nih.gov/sites/entrez?cmd=Retrieve&amp;db=PubMed&amp;list_uids=24801599&amp;dopt=Abstract) | Corneal dystrophy, granular type II |
|  |  |  |  | [Weiss (2008) Cornea 27S2: S1 PubMed: 19337156](http://www.ncbi.nlm.nih.gov/sites/entrez?cmd=Retrieve&amp;db=PubMed&amp;list_uids=19337156&amp;dopt=Abstract) | Variant lattice corneal dystrophy. IC3D classification. |
| p.A620D | NM_000358.2:c.1859C>A | Corneal dystrophy, lattice type | Asia | [Lakshminarayanan (2011) Br J Ophthalmol 95, 1457](http://www.ncbi.nlm.nih.gov/sites/entrez?cmd=Retrieve&amp;db=PubMed&amp;list_uids=21835759&amp;dopt=Abstract) | Primary literature report |
|  |  |  |  | [Elavazhagan (2012) Protein Expr Purif 84: 108 [Functional characterisation]](http://www.ncbi.nlm.nih.gov/sites/entrez?cmd=Retrieve&amp;db=PubMed&amp;list_uids=22575305&amp;dopt=Abstract) | Reduced solubility and increased abundance in  inclusion bodies compared to wild-type. |
|  |  |  |  | Hao (2016) Int J Ophthalmol 9(2):198 PubMed: 26949635 |  |
| p.A620P | NM_000358.2:c.1858G>C | Corneal dystrophy, lattice type IIIA | Asia | [Jung (2014) Cornea 33, 1324](http://www.ncbi.nlm.nih.gov/sites/entrez?cmd=Retrieve&amp;db=PubMed&amp;list_uids=25321938&amp;dopt=Abstract) | Primary literature report |
| p.T621P | NM_000358.2:c.1861A>C | Corneal dystrophy, lattice type I | Asia | [Song (2015) Ann Lab Med 35, 336](http://www.ncbi.nlm.nih.gov/sites/entrez?cmd=Retrieve&amp;db=PubMed&amp;list_uids=25932442&amp;dopt=Abstract) | Primary literature report |
|  |  |  |  | [Lee (2016) J Refract Surg 32: 356 [Additional phenotype]](http://www.ncbi.nlm.nih.gov/sites/entrez?cmd=Retrieve&amp;db=PubMed&amp;list_uids=27163623&amp;dopt=Abstract) | Corneal dystrophy, lattice type IIIA |
| p.N622H | NM_000358.2:c.1864A>C | Corneal dystrophy, lattice intermediate type I/IIIA | Europe | [Stewart (1999) Ophthalmology 106: 964 PubMed: 10328397](http://www.ncbi.nlm.nih.gov/sites/entrez?cmd=Retrieve&amp;db=PubMed&amp;list_uids=10328397&amp;dopt=Abstract) | Primary literature report |
|  |  |  |  | Anandalakshmi (2017) Biochem J 474: 1705 PubMed: 28381645 |  |
|  |  |  |  | [Munier (2002) Invest Ophthalmol Vis Sci 43: 949 PubMed: 11923233](http://www.ncbi.nlm.nih.gov/sites/entrez?cmd=Retrieve&amp;db=PubMed&amp;list_uids=11923233&amp;dopt=Abstract) | Genotype-phenotype correlation. |
|  |  |  |  | [Weiss (2008) Cornea 27S2: S1 PubMed: 19337156](http://www.ncbi.nlm.nih.gov/sites/entrez?cmd=Retrieve&amp;db=PubMed&amp;list_uids=19337156&amp;dopt=Abstract) | Variant lattice corneal dystrophy. IC3D classification. |
| p.N622K | NM_000358.2:c.1866T>A | Corneal dystrophy, lattice type IIIA | Europe | [Munier (2002) Invest Ophthalmol Vis Sci 43, 949](http://www.ncbi.nlm.nih.gov/sites/entrez?cmd=Retrieve&amp;db=PubMed&amp;list_uids=11923233&amp;dopt=Abstract) | Primary literature report |
|  |  |  |  | [Weiss (2008) Cornea 27S2: S1 [Additional report]](http://www.ncbi.nlm.nih.gov/sites/entrez?cmd=Retrieve&amp;db=PubMed&amp;list_uids=19337156&amp;dopt=Abstract) | Variant lattice corneal dystrophy. IC3D classification. |
| p.N622K | NM_000358.2:c.1866T>G | Corneal dystrophy, lattice type IIIA | Europe | [Munier (2002) Invest Ophthalmol Vis Sci 43, 949](http://www.ncbi.nlm.nih.gov/sites/entrez?cmd=Retrieve&amp;db=PubMed&amp;list_uids=11923233&amp;dopt=Abstract) | Primary literature report |
|  |  |  |  | [Weiss (2008) Cornea 27S2: S1 [Additional report]](http://www.ncbi.nlm.nih.gov/sites/entrez?cmd=Retrieve&amp;db=PubMed&amp;list_uids=19337156&amp;dopt=Abstract) | Variant lattice corneal dystrophy. IC3D classification. |
| p.G623R | NM_000358.2:c.1867G>C | Corneal dystrophy, map-like | Europe | [Gruenauer-Kloevekorn (2009) Graefes Arch Clin Exp](http://www.ncbi.nlm.nih.gov/sites/entrez?cmd=Retrieve&amp;db=PubMed&amp;list_uids=18777038&amp;dopt=Abstract)  [Ophthalmol 247: 93 PubMed: 18777038](http://www.ncbi.nlm.nih.gov/sites/entrez?cmd=Retrieve&amp;db=PubMed&amp;list_uids=18777038&amp;dopt=Abstract) | Primary literature report |
|  |  |  |  | Anandalakshmi (2017) Biochem J 474: 1705 PubMed: 28381645 |  |
| p.G623D | NM_000358.2:c.1868G>A | Corneal dystrophy, lattice intermediate type I/IIIA | Asia, Europe, North America | [Afshari (2001) Arch Ophthalmol 119: 16 PubMed: 11146721](http://www.ncbi.nlm.nih.gov/entrez/query.fcgi?cmd=Retrieve&amp;db=PubMed&amp;list_uids=11146721&amp;dopt=Abstract) | Primary literature report |
|  |  |  |  | [Aldave (2005) Ophthalmology 112: 1017 PubMed: 15885785](http://www.ncbi.nlm.nih.gov/sites/entrez?cmd=Retrieve&amp;db=PubMed&amp;list_uids=15885785&amp;dopt=Abstract) | Corneal dystrophy, Bowman type |
|  |  |  |  | [Anandalakshmi (2017) Biochem J 474: 1705 PubMed: 28381645](http://www.ncbi.nlm.nih.gov/sites/entrez?cmd=Retrieve&amp;db=PubMed&amp;list_uids=28381645&amp;dopt=Abstract) |  |
|  |  |  |  | Auw-Haedrich (2009) Ophthalmol 116(1):46 PubMed: 19019446 |  |
|  |  |  |  | [Evans (2016) Invest Ophthalmol Vis Sci 57: 5407 PubMed: 27737463](http://www.ncbi.nlm.nih.gov/sites/entrez?cmd=Retrieve&amp;db=PubMed&amp;list_uids=27737463&amp;dopt=Abstract) | Corneal dystrophy, epithelial basement membrane |
|  |  |  |  | [Li (2008) Mol Vis 14: 1298 PubMed: 18636123](http://www.ncbi.nlm.nih.gov/sites/entrez?cmd=Retrieve&amp;db=PubMed&amp;list_uids=18636123&amp;dopt=Abstract) | Corneal dystrophy, Reis-Buckler |
|  |  |  |  | [Munier (2002) Invest Ophthalmol Vis Sci 43: 949 PubMed: 11923233](http://www.ncbi.nlm.nih.gov/sites/entrez?cmd=Retrieve&amp;db=PubMed&amp;list_uids=11923233&amp;dopt=Abstract) | Genotype-phenotype correlation. |
|  |  |  |  | [Weiss (2008) Cornea 27S2: S1 PubMed: 19337156](http://www.ncbi.nlm.nih.gov/sites/entrez?cmd=Retrieve&amp;db=PubMed&amp;list_uids=19337156&amp;dopt=Abstract) | Variant lattice corneal dystrophy. IC3D classification. |
| p.V624M | NM_000358.2:c.1870G>A | Corneal dystrophy, lattice type | Asia, Europe, North America | [Kannabiran (2006) Hum Mutat 27: 615 PubMed: 16683255](http://www.ncbi.nlm.nih.gov/sites/entrez?cmd=Retrieve&amp;db=PubMed&amp;list_uids=16683255&amp;dopt=Abstract) | Primary literature report |
|  |  |  |  | Afshari (2008) Mol Vis 14: 495 PubMed: 18385782 |  |
|  |  |  |  | Anandalakshmi (2017) Biochem J 474: 1705 PubMed: 28381645 | Functional characterisation |
|  |  |  |  | [Karring (2012) Exp Eye Res 96: 163 PubMed: 22155582](http://www.ncbi.nlm.nih.gov/sites/entrez?cmd=Retrieve&amp;db=PubMed&amp;list_uids=22155582&amp;dopt=Abstract) |  |
| p.V625D | NM_000358.2:c.1874T>A | Corneal dystrophy, lattice type | Asia | [Tian (2007) Am J Ophthalmol 144, 473](http://www.ncbi.nlm.nih.gov/sites/entrez?cmd=Retrieve&amp;db=PubMed&amp;list_uids=17765440&amp;dopt=Abstract) | Primary literature report |
|  |  |  |  | Anandalakshmi (2017) Biochem J 474: 1705 PubMed: 28381645 |  |
|  |  |  |  | [Weiss (2008) Cornea 27S2: S1 [Additional report]](http://www.ncbi.nlm.nih.gov/sites/entrez?cmd=Retrieve&amp;db=PubMed&amp;list_uids=19337156&amp;dopt=Abstract) | Variant lattice corneal dystrophy. IC3D classification. |
| p.H626R | NM_000358.2:c.1877A>G | Corneal dystrophy, lattice intermediate type I/IIIA | Asia, Europe, North America and South America | [Stewart (1999) Ophthalmology 106: 964 PubMed: 10328397](http://www.ncbi.nlm.nih.gov/entrez/query.fcgi?cmd=Retrieve&amp;db=PubMed&amp;list_uids=10328397&amp;dopt=Abstract) | Primary literature report |
|  |  |  |  | [Anandalakshmi (2017) Biochem J 474: 1705 PubMed: 28381645](http://www.ncbi.nlm.nih.gov/sites/entrez?cmd=Retrieve&amp;db=PubMed&amp;list_uids=28381645&amp;dopt=Abstract) |  |
|  |  |  |  | [Cai (2016) Genet Test Mol Biomarkers 20: 388 PubMed: 27348782](http://www.ncbi.nlm.nih.gov/sites/entrez?cmd=Retrieve&amp;db=PubMed&amp;list_uids=27348782&amp;dopt=Abstract) |  |
|  |  |  |  | Chau (2003) Br J Ophthalmol 87(6):686 PubMed: 12770961 |  |
|  |  |  |  | [Elavazhagan (2012) Protein Expr Purif 84: 108 PubMed: 22575305](http://www.ncbi.nlm.nih.gov/sites/entrez?cmd=Retrieve&amp;db=PubMed&amp;list_uids=22575305&amp;dopt=Abstract) | Solubility and secondary structure as wild-type.  Increased stability comp. to wild-type. |
|  |  |  |  | Hao (2016) Int J Ophthalmol 9(2):198 PubMed: 26949635 |  |
|  |  |  |  | [Lai (2014) Cornea 33: 726 PubMed: 24831201](http://www.ncbi.nlm.nih.gov/sites/entrez?cmd=Retrieve&amp;db=PubMed&amp;list_uids=24831201&amp;dopt=Abstract) |  |
|  |  |  |  | [Munier (2002) Invest Ophthalmol Vis Sci 43: 949 PubMed: 11923233](http://www.ncbi.nlm.nih.gov/sites/entrez?cmd=Retrieve&amp;db=PubMed&amp;list_uids=11923233&amp;dopt=Abstract) | Genotype-phenotype correlation. |
|  |  |  |  | Nowinska (2011) Mol Vis 17:2333 PubMed: 21921985 |  |
|  |  |  |  | Schmitt–Bernard (2000) Invest Ophthalmol Vis Sci 41(6):1302 PubMed:  10798644 |  |
|  |  |  |  | [Wang (2013) Eye Sci 28: 144 PubMed: 24579556](http://www.ncbi.nlm.nih.gov/sites/entrez?cmd=Retrieve&amp;db=PubMed&amp;list_uids=24579556&amp;dopt=Abstract) | Corneal dystrophy, lattice type IIIB |
|  |  |  |  | [Wang (2017) Mol Med Rep 15: 3198 PubMed: 28358433](http://www.ncbi.nlm.nih.gov/sites/entrez?cmd=Retrieve&amp;db=PubMed&amp;list_uids=28358433&amp;dopt=Abstract) |  |
|  |  |  |  | [Weiss (2008) Cornea 27S2: S1 PubMed: 19337156](http://www.ncbi.nlm.nih.gov/sites/entrez?cmd=Retrieve&amp;db=PubMed&amp;list_uids=19337156&amp;dopt=Abstract) | Variant lattice corneal dystrophy. IC3D classification. |
|  |  |  |  | [Yang (2010) Mol Vis 16: 1186 PubMed: 20664689](http://www.ncbi.nlm.nih.gov/sites/entrez?cmd=Retrieve&amp;db=PubMed&amp;list_uids=20664689&amp;dopt=Abstract) |  |
|  |  |  |  | [Zenteno (2006) Arch Soc Esp Oftalmol 81: 369 PubMed: 16888689](http://www.ncbi.nlm.nih.gov/sites/entrez?cmd=Retrieve&amp;db=PubMed&amp;list_uids=16888689&amp;dopt=Abstract) | Corneal dystrophy, granular type |
| p.H626P | NM_000358.2:c.1877A>C | Corneal dystrophy, lattice intermediate type I/IIIA | Asia, Europe, New Zealand and South America | [Munier (2002) Invest Ophthalmol Vis Sci 43: 949 PubMed: 11923233](http://www.ncbi.nlm.nih.gov/sites/entrez?cmd=Retrieve&amp;db=PubMed&amp;list_uids=11923233&amp;dopt=Abstract) | Primary literature report |
|  |  |  |  | Anandalakshmi (2017) Biochem J 474: 1705 PubMed: 28381645 |  |
|  |  |  |  | Liskova (2008) Ophthalmic Res 40: 105 PubMed: 18259096 | Superficial geographic corneal opacities |
|  |  |  |  | Vincent (2010) Br J Ophthalmol 94: 836 PubMed: 19948560 | Corneal dystrophy, Bowman layer |
|  |  |  |  | Weiss (2008) Cornea 27S2: S1 PubMed: 19337156 |  |
|  |  |  |  | [Wheeldon (2008) Mol Vis 14:1503 PubMed: 18728790](https://www.ncbi.nlm.nih.gov/pubmed/?term=18728790) | Variant lattice corneal dystrophy. IC3D classification. |
| p.V631D | NM_000358.2:c.1892T>A | Corneal dystrophy, lattice type, with deep deposits | Europe | [Munier (2002) Invest Ophthalmol Vis Sci 43: 949 PubMed: 11923233](http://www.ncbi.nlm.nih.gov/sites/entrez?cmd=Retrieve&amp;db=PubMed&amp;list_uids=11923233&amp;dopt=Abstract) | Primary literature report |
|  |  |  |  | [Anandalakshmi (2017) Biochem J 474: 1705 PubMed: 28381645](http://www.ncbi.nlm.nih.gov/sites/entrez?cmd=Retrieve&amp;db=PubMed&amp;list_uids=23455751&amp;dopt=Abstract) |  |
|  |  |  |  | Laborante (2013) Clin Ter 164: e41 PubMed: 23455751 | Lattice corneal dystrophy, type I |
|  |  |  |  | [Weiss (2008) Cornea 27S2: S1 PubMed: 19337156](http://www.ncbi.nlm.nih.gov/sites/entrez?cmd=Retrieve&amp;db=PubMed&amp;list_uids=19337156&amp;dopt=Abstract) | Variant lattice corneal dystrophy. IC3D classification. |
| p.R666S | NM_000358.2:c.1998G>C | Corneal dystrophy, epithelial basement membrane | Asia and Europe | [Boutboul (2006) Hum Mutat 27, 553](http://www.ncbi.nlm.nih.gov/sites/entrez?cmd=Retrieve&amp;db=PubMed&amp;list_uids=16652336&amp;dopt=Abstract) | Primary literature report |
|  |  |  |  | [Weiss (2008) Cornea 27S2: S1 [Additional report]](http://www.ncbi.nlm.nih.gov/sites/entrez?cmd=Retrieve&amp;db=PubMed&amp;list_uids=19337156&amp;dopt=Abstract) | IC3D classification. |
|  |  |  |  | [Xiong (2015) Science 347: 1254806 [Additional report]](http://www.ncbi.nlm.nih.gov/sites/entrez?cmd=Retrieve&amp;db=PubMed&amp;list_uids=25525159&amp;dopt=Abstract) | predicted to induce a large splicing change - Table S4. |
| N/A | N/A | Corneal granular dystrophy | Europe and North America | [Rodrigues (1975) Trans Am Ophthalmol Soc 73:306 PMC1311459](https://www.ncbi.nlm.nih.gov/pmc/articles/PMC1311459/) |  |
|  |  |  |  | [Tripathi (1970) Br J Ophthalmol 54(6):361 PubMed: 4915170](https://www.ncbi.nlm.nih.gov/pubmed/4915170) |  |
| N/A | N/A | Granular corneal dystrophy | Europe | Lyons (1994) Ophthalmol 101(11):1812 PubMed: 7800362 |  |
|  |  |  |  | [Severin (1998) Graefes Arch Clin Exp Ophthalmol 236(4):291 PubMed:](https://www.ncbi.nlm.nih.gov/pubmed/9561363)  [9561363](https://www.ncbi.nlm.nih.gov/pubmed/9561363) |  |
|  |  |  |  | Maeng (2017) Yonsei Med 58(2):423 PubMed: 28120575 |  |
| N/A | N/A | Reis-Bücklers' dystrophy | Europe | [Rice (1968) Br J Ophthalmol 52(8):577 PMC506649](https://www.ncbi.nlm.nih.gov/pmc/articles/PMC506649/) |  |
| N/A | N/A | *TGFBI* | North America | [Weissman (2015) J Refract Surg 31(1):61 PubMed: 25420001](https://www.ncbi.nlm.nih.gov/pubmed/?term=25420001) |  |

| Total HGMD paper count |
| --- |
| PubMed paper count |
| Total paper count |

| 120 |
| --- |
| 64 |
| 184 |
